# Transcriptome Analysis Reveals Differential Gene Expression between the Closing Ductus Arteriosus and the Patent Ductus Arteriosus in Humans

**DOI:** 10.3390/jcdd8040045

**Published:** 2021-04-16

**Authors:** Junichi Saito, Tomoyuki Kojima, Shota Tanifuji, Yuko Kato, Sayuki Oka, Yasuhiro Ichikawa, Etsuko Miyagi, Tsuyoshi Tachibana, Toshihide Asou, Utako Yokoyama

**Affiliations:** 1 Department of Physiology, Tokyo Medical University, 6-1-1 Shinjuku, Shinjuku-ku, Tokyo 160-8402, Japan; junichi.saito@yale.edu (J.S.); kojimat@tokyo-med.ac.jp (T.K.); tanifuji@tokyo-med.ac.jp (S.T.); yukato@tokyo-med.ac.jp (Y.K.); s120052@tokyo-med.ac.jp (S.O.); 2 Department of Obstetrics and Gynecology, Yokohama City University, 3-9 Fukuura, Kanazawa-ku, Yokohama, Kanagawa 236-0004, Japan; emiyagi@yokohama-cu.ac.jp; 3 Department of Cardiovascular Surgery, Kanagawa Children’s Medical Center, 2-138-4 Mutsukawa, Minami-ku, Yokohama, Kanagawa 232-8555, Japan; yas1lll@yahoo.co.jp (Y.I.); tachi.t@icloud.com (T.T.); toshisurgeonfamily@live.jp (T.A.)

**Keywords:** ductus arteriosus, neointima, tunica media, congenital heart disease, transcriptome, neural crest

## Abstract

The ductus arteriosus (DA) immediately starts closing after birth. This dynamic process involves DA-specific properties, including highly differentiated smooth muscle, sparse elastic fibers, and intimal thickening (IT). Although several studies have demonstrated DA-specific gene expressions using animal tissues and human fetuses, the transcriptional profiles of the closing DA and the patent DA remain largely unknown. We performed transcriptome analysis using four human DA samples. The three closing DA samples exhibited typical DA morphology, but the patent DA exhibited aorta-like elastic lamellae and poorly formed IT. A cluster analysis revealed that samples were clearly divided into two major clusters, the closing DA and patent DA clusters, and showed distinct gene expression profiles in IT and the tunica media of the closing DA samples. Cardiac neural crest-related genes such as *JAG1* were highly expressed in the tunica media and IT of the closing DA samples compared to the patent DA sample. Abundant protein expressions of jagged 1 and the differentiated smooth muscle marker calponin were observed in the closing DA samples but not in the patent DA sample. Second heart field-related genes such as *ISL1* were enriched in the patent DA sample. These data indicate that the patent DA may have different cell lineages compared to the closing DA.

## 1. Introduction

The ductus arteriosus (DA) is a fetal vascular shunt that connects the pulmonary artery and the aorta, and it is essential for maintaining fetal circulation. The DA begins to close immediately after birth. This closing process is characterized by several DA-specific features including differentiated contractile smooth muscle cells (SMCs), fragmentation of internal elastic laminae, sparse elastic fiber formation in the tunica media, and intimal thickening (IT) formation [1,2,3,4,5,6]. Histological analyses of human DA samples and several animal models indicated that these DA-specific features gradually develop throughout the fetal and neonatal periods [7]. Patent DA is a condition where the DA does not close properly after birth. Patent DA occurs in approximately 1 in 2000 full-term infants and occurs more frequently in premature neonates [8]. PDA samples exhibit fewer DA-specific structural features [9]. Comprehensive analysis of gene expression comparing the closing DA and the patent DA is necessary in order to better understand DA-specific remodeling and to explore methods to regulate patency of the DA.

Several research groups have previously reported the gene expression profiles of mouse, rat, and ovine DAs [10,11,12,13,14,15,16,17]. However, transcriptome analyses of human DA tissues are limited [18,19]. Yarboro et al. performed RNA sequencing using human DA tissues of 21 weeks gestation and identified genes that were distinctly expressed in the DA tissue compared to the aorta [18]. Additionally, they compared their human RNA sequencing data with previously reported rodent microarray data and demonstrated transcriptional commonalities between human and rodent DAs [18]. A report by Mueller et al. [19] is presently the only published study that demonstrated the differences of transcriptional profiles using postnatal human DAs. They compared gene expression between stent-implanted open DAs on postnatal days 222 and 239, closed ligamentous DAs on day 147, and an open unstented DA on day 1. To the best of our knowledge, transcriptomic comparisons of the closing DA and the patent DA in human infants have not been previously reported.

Using four postnatal human DA tissues, we performed an unbiased transcriptome analysis using the IT and the tunica media of each DA tissue. We found differential gene expression between the tunica media of the closing DA and that of the patent DA. Additionally, we investigated genes enriched in the IT or the tunica media of closing DA tissues to identify genes that potentially contribute to DA-specific remodeling.

## 2. Materials and Methods

### 2.1. Study Subjects and Ethics Statements

The protocol for using human DA tissues was approved by the Research Ethics Committees at Tokyo Medical University and Kanagawa Children’s Medical Center (reference numbers: T2020-0238 and 1502-05, respectively). The protocol conformed to the principles outlined in the Declaration of Helsinki. After receiving written informed parental consent, human DA tissues were obtained from four patients with congenital heart diseases, during cardiac surgeries in Kanagawa Children’s Medical Center. The patient information for each of the four cases included in this study is summarized in Table 1.

### 2.2. Total RNA Preparation and Microarray Analysis

**Figure 1:**
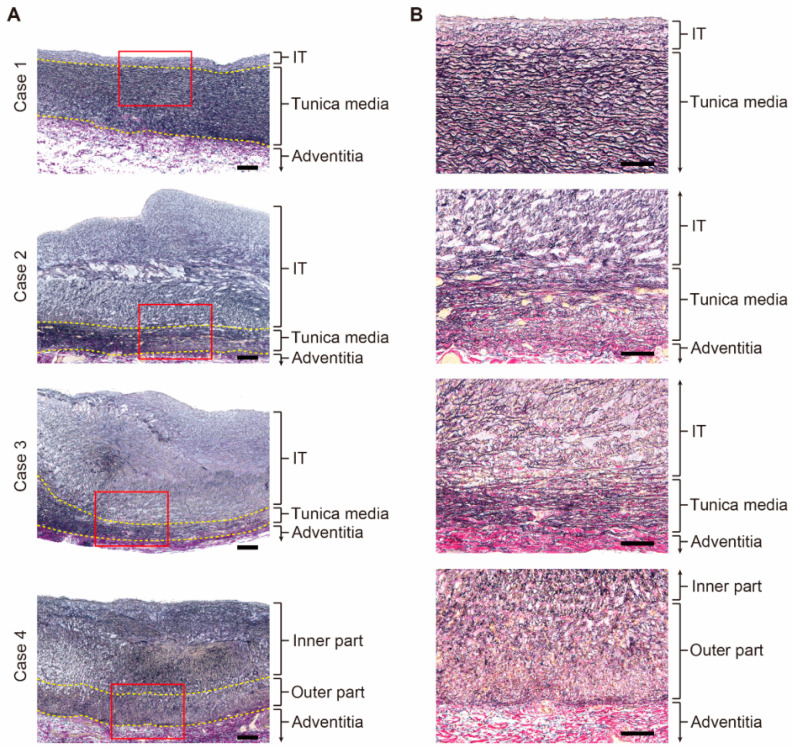
Histological analysis of the human ductus arteriosus (DA) tissues. (**A**) Lower magnification images of the Elastica van Gieson stain of the human DA tissues. The yellow dotted lines indicate the border between the intimal thickening (IT) and the tunica media and the border between the tunica media and the adventitia. Scale bars: 200 µm. (**B**) Magnified images of red boxes in (**A**). Scale bars: 100 µm.

Four human DA tissues were subjected to tissue staining and transcriptome analysis. Each DA tissue was divided into two pieces. One piece was fixed with 10% buffered formalin (FUJIFILM Wako Pure Chemical Corporation, Osaka, Japan) for tissue staining. The other piece of tissue was prepared for microarray analysis as follows. After the adventitia was removed, each DA tissue was divided into two parts: the inner part and the outer part, which mainly contained IT and the tunica media, respectively, as indicated by the yellow dotted lines in Figure 1A. The tissues were immediately frozen in liquid nitrogen and stored at −80 °C until all patient samples were collected. Total RNA preparation and microarray analysis were performed as described previously [10,11]. Briefly, the frozen tissues were disrupted by a multi-bead shocker instrument (Yasui Kikai, Osaka, Japan). After buffer RLT with β-mercaptoethanol was added to the tissues, they were sonicated to ensure the samples were uniformly homogeneous. Total RNA was isolated using a RNeasy Mini Kit (Qiagen, Venlo, The Netherlands). Microarray experiments were carried out using a SurePrint G3 Human GE 8 × 60 K v2 Microarray (Agilent, Santa Clara, CA, USA) according to the manufacturer’s protocol.

### 2.3. Generation of a Dendrogram, Venn Diagrams, and a Heatmap

The dendrogram was generated with Ward’s method using the hclust and dendrogram functions in R. The packages gplots and genefilter in R were used to create a heatmap in which data was normalized into a *z*-score. The mapping grids were subsequently colored according to their *z*-score. Venn diagrams of the number of differentially expressed genes in each sample group were generated using the gplots package in R.

### 2.4. Gene Set Enrichment Analyses (GSEAs)

Gene set enrichment analyses (GSEAs) were conducted to investigate the functions of genes that significantly correlated with each sample group. GSEAs ranked the gene list by the correlation between genes and phenotype, and an enrichment score was calculated to assess the gene distribution. Each analysis was carried out with 1000 permutations. Gene sets were considered significantly enriched if the false discovery rate (FDR) *q*-value < 0.25 [20].

### 2.5. Tissue Staining and Immunohistochemistry

Paraffin-embedded blocks containing the human DA tissues were cut into 4 μm thick sections and placed on glass slides. Elastica van Gieson staining was performed for morphological analysis to evaluate IT and the tunica media, as described previously [21,22]. Immunohistochemistry was performed using primary antibodies for jagged 1 (sc-390177, Santa Cruz Biotechnology, Dallas, TX, USA) and calponin (M3556, DakoCytomation, Glostrup, Denmark). Biotinylated rabbit antibody (Vectastain Elite ABC IgG kit, Vector Labs, Burlingame, CA, USA) was used as a secondary antibody, and the presence of targeted proteins was determined by 3,3′-diaminobenzidine tetrahydrochloride (DAB) (DakoCytomation, Glostrup, Denmark). Negative staining of immunohistochemistry was confirmed by the omission of primary antibodies.

## 3. Results

### 3.1. DA-Related Clinical Course of Each Participant

Four patients with congenital heart diseases were analyzed in this study. Patient profiles are presented in Table 1. Case 1 was considered a patent DA case because the DA did not exhibit closing tendency throughout the clinical course. The DA tissue was isolated during an operation for an atrioventricular septal defect closure and repairs of the aortic arch and pulmonary venous returns. The other three cases (Cases 2–4) exhibited complex congenital heart diseases that required DA patency to maintain systemic circulation. Cases 2–4 were administered prostaglandin E1 (PGE_1_) because they exhibited closing tendency of the DA.

In Case 2, lipo-PGE_1_ (1 ng/kg/min) was administered 8 h after birth when an echocardiography indicated narrowing of the DA. Case 2 continued lipo-PGE_1_ treatment until the operation. The patient had an aortic repair and pulmonary artery banding (PAB) conducted on postnatal day 5.

Case 3 showed closing tendency of the DA soon after birth and was administrated lipo-PGE_1_ (2 ng/kg/min). The dose of lipo-PGE_1_ was increased (4 ng/kg/min) 8 h after birth due to further closing tendency. The patient had PAB conducted on postnatal day 3. The closing tendency of the DA remained and required PGE_1_-cyclodextrin (30 ng/kg/min) on postnatal day 4. The patient received PGE_1_-cyclodextrin until the Norwood operation was conducted on postnatal day 24.

Case 4 showed closing tendency of the DA at 9 h after birth and received a lipo-PGE_1_ infusion (1 ng/kg/min). The patient underwent PAB on postnatal day 3. The DA was gradually narrowed and required an increased dose of lipo-PGE_1_ (5 ng/kg/min) on postnatal day 70. The patient underwent the Norwood operation on postnatal day 98. On the basis of their clinical courses, Cases 2–4 were considered as closing DAs.

### 3.2. Histological Differences between the Patent DA and the Closing DA Tissues

The Elastica van Gieson stain demonstrated that Case 1 had well-organized layered elastic fibers in the tunica media and a poorly formed IT (Figure 1A, upper panel). In Case 1, there was no overt fragmentation of the internal elastic laminae (Figure 1B, upper panel). Case 2 and Case 3 showed prominent IT formation that protruded into the lumen (Figure 1A, middle panels). Circumferentially oriented layered elastic fibers in the tunica media were sparsely formed and the internal elastic laminae were highly fragmented (Figure 1B, middle panels). Similarly, Case 4, who received PGE_1_ administration for more than 3 months, had a prominent IT (Figure 1A, lower panel). However, the entire tunica media consisted of sparse elastic fibers radially oriented toward the internal lumen, and circumferentially oriented elastic fibers were not recognized (Figure 1B, lower panel). These findings indicated that the closing DA had well-recognized, DA-specific morphological features, including prominent IT formation, fragmented internal elastic laminae, and less elastic fibers in the tunica media, which seemed to reflect a normal closing process. On the other hand, the patent DA tissue (Case 1) was devoid of these structures and exhibited aortification of the vascular wall, which was consistent with previously reported morphological characteristics of the patent DA [9].

### 3.3. Microarray Analysis of the IT and the Tunica Media of Human DA Tissues

To elucidate a differential gene expression profile between the patent DA (Case 1) and the closing DA tissues (Cases 2–4), we performed an unbiased transcriptomic analysis using these human DA tissues. Each DA sample was divided into the IT and the tunica media in Cases 1–3. In Case 4, circumferentially oriented SMCs and layered elastic laminae could not be identified; therefore, the IT-like wall was divided into the inner part and the outer part (Figure 1A, lower). These samples were subjected to microarray analysis.

**Figure 2:**
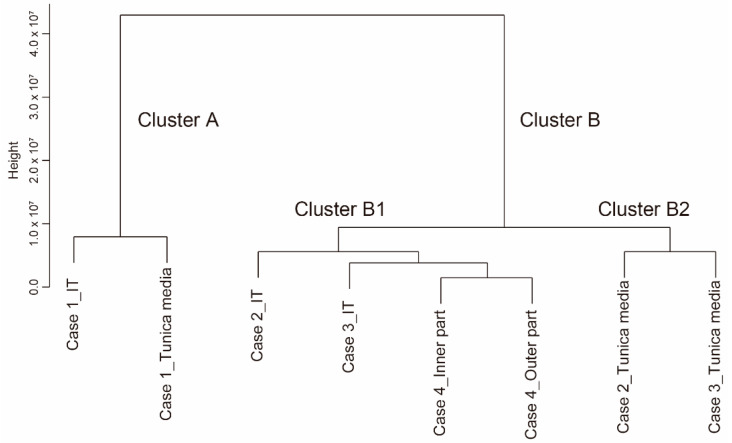
A dendrogram of the gene expressions from human ductus arteriosus tissues. IT: intimal thickening.

The dendrogram demonstrated that the human DA tissues were clearly divided into two major clusters, A and B (Figure 2). Cluster A consisted of both the IT and the tunica media from Case 1. Cluster B consisted of the samples from Cases 2–4, and this cluster was further divided into two subgroups, B1 and B2. Cluster B1 consisted of the IT tissues from Cases 2 and 3 and both the inner and outer IT-like parts from Case 4. Cluster B2 consisted of the tunica media samples from Cases 2 and 3. These data suggested that the patent DA tissue (Case 1) had a distinct gene expression pattern compared to the other closing DA samples (Cases 2–4). In Cases 2 and 3, the gene expression patterns of the tunica media samples were relatively similar. Additionally, the IT samples showed similar gene expression profiles, which were distant from the tunica media samples of Cases 2 and 3. In agreement with histological analysis showing that two parts of the DA tissue from Case 4 (inner and outer parts) exhibited an IT-like structure, these two samples of Case 4 showed similar gene patterns, which were close to that of the IT of Cases 2 and 3.

### 3.4. Transcriptomic Differences between the Tunica Media of Closing DA Tissues and the Patent DA Tissue

Both the histological assessment and the cluster analysis of DA tissues demonstrated that the gene expression profile of the tunica media of the patent DA tissue (Case 1) was markedly different from that of the closing DA tissues (Cases 2 and 3). We, thus, compared gene expressions between the tunica media of the patent DA and closing DA tissues.

**Figure 3:**
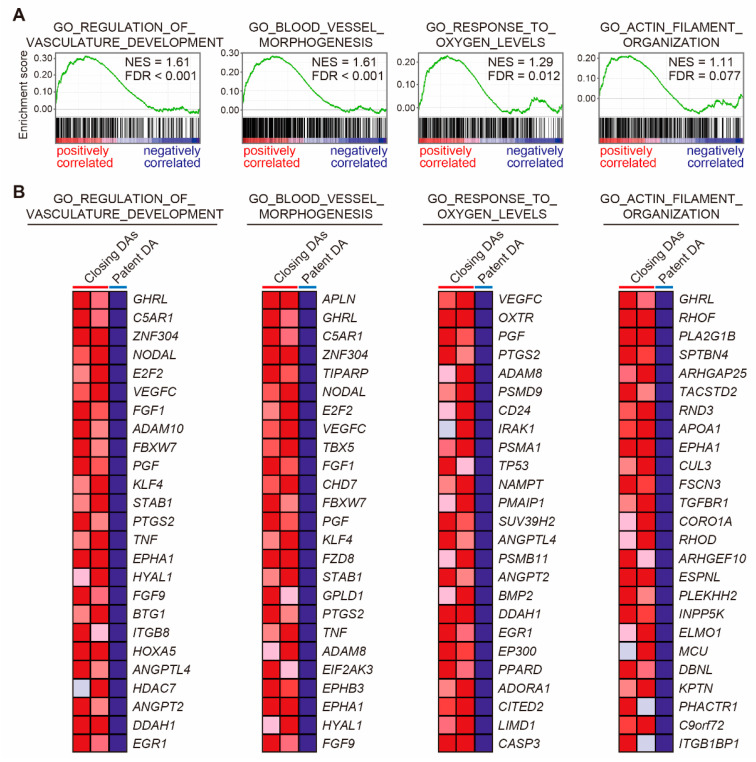
Gene set enrichment analyses (GSEAs) of the tunica media of the closing human ductus arteriosus (DA) and the patent DA tissues. (**A**) GSEAs revealed positive correlations between the tunica media of the closing human DA tissues and vascular development–related genes, oxygen level response-related genes, and actin filament organization-related genes. On the *x*-axis, the genes in each gene set are ranked from the left side (positively correlated) to the right side (negatively correlated). The vertical black lines that look like barcodes indicate each gene in the gene set. The *y*-axis displays the calculated enrichment score of each gene (green color). NES, normalized enrichment score; FDR, false discovery rate. (**B**) The top 25 genes that comprise the leading edge of the enrichment score in (**A**) are shown in each heatmap.

The GSEAs between the tunica media of the closing DA and the patent DA tissues, using all gene sets related to biological processes in the Gene Ontology (GO) (size > 300), revealed that the closing DA tissues were significantly correlated to 87 biological processes (FDR < 0.25, Table 2). Notably, vascular development-related gene sets (GO_REGULATION_OF_VASCULATURE_DEVELOPMENT and GO_BLOOD_VESSEL_MORPHOGENESIS) were highly enriched in the tunica media of closing DA tissues (Figure 3). Kinase activation-related gene sets (GO_REGULATION_OF_MAP_KINASE_ACTIVITY, GO_ACTIVATION_OF_PROTEIN_KINASE_ACTIVITY, and GO_POSITIVE_REGULATION_OF_PROTEIN_SERINE_THREONINE_KINASE_ACTIVITY) and three catabolic process-related gene sets, including GO_REGULATION_OF_PROTEIN_CATABOLIC_PROCESS, were positively correlated with the closing DA tunica media tissue. This suggested that intracellular signaling was more actively regulated in the closing DA tissues compared to the patent DA tissue. Protein secretion-related gene sets (GO_POSITIVE_REGULATION_OF_SECRETION and GO_GOLGI_VESICLE_TRANSPORT) and adhesion-related gene sets (GO_POSITIVE_REGULATION_OF_CELL_ADHESION, GO_REGULATION_OF_CELL_CELL_ADHESION, and GO_CELL_SUBSTRATE_ADHESION) were also enriched in the closing DA tissues, which support previous reports which found that multiple extracellular matrices and cell–matrix interactions play roles in DA-specific physiological remodeling [5,21,22]. The gene set GO_RESPONSE_TO_OXYGEN_LEVELS was positively correlated to the closing DA tissues (Figure 3). In this gene set, *EGR1*, which was previously shown to increase immediately after birth in rat DA tissues [10], was upregulated in the tunica media of the closing DA tissues. Enrichment of the gene set GO_ACTIN_FILAMENT_ORGANIZATION in the closing DA tissues (Figure 3) contained the Rho GTPase *RHOD*, which regulates directed cell migration [23]. This may support the migratory feature of SMCs in the closing DA tissue.

Although we demonstrated several genes that were highly expressed in the tunica media of the closing DAs compared to that of the patent DA (Figure 3, and Table 2 and Table 3), postnatal PGE_1_ administration possibly affected these gene expressions of the tunica media. To address this issue, we compared gene expressions of the tunica media between shorter-term PGE_1_-treated DAs (less than one month of administration, Cases 2 and 3) and a longer-term PGE_1_-treated DA (more than three months of administration, Case 4). The GSEAs revealed that the outer part of longer-term PGE_1_-treated DA was significantly correlated to eight biological processes related to cell-cycle regulation (FDR < 0.25, Table S1 and Figure S1A,B, Supplementary Materials) compared to the tunica media of shorter-term PGE_1_-treated DAs. Among these gene sets, the gene sets GO_ORGANELLE_FISSION and GO_NEGATIVE_REGULATION_OF_CELL_CYCLE_PROCESS belonged to gene sets that were highly expressed in the tunica media of the closing DAs (Table 2). These two gene sets may be associated with PGE_1_ administration, but not with specific features of the closing DA. However, the remaining 85 gene sets in Table 2 seemed to be independent of duration of PGE_1_ administration.

### 3.5. Vascular Development-Related Genes in Human DA Tissues

**Figure 4:**
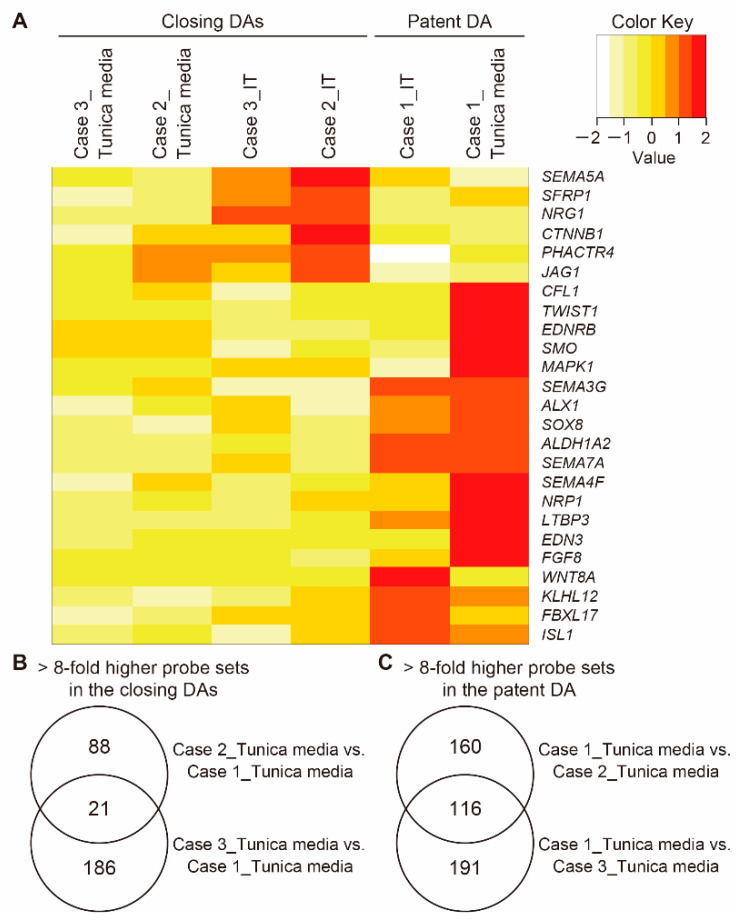
Differential gene expression between the tunica media of the closing human ductus arteriosus (DA) tissues and that of the patent DA tissue. (**A**) A heatmap of vascular cell lineage-related genes is depicted. (**B**) A Venn diagram shows the number of probe sets that were highly expressed (>8-fold) in the tunica media of closing DA tissues (Cases 2 and 3) compared to that of the patent DA tissue (Case 1). (**C**) A Venn diagram shows the number of probe sets highly expressed (>8-fold) in the tunica media of the patent DA tissue (Case 1) compared to that of the closing DA tissues (Cases 2 and 3). IT, intimal thickening.

The vascular development-related gene sets noted above (Table 2 and Figure 3) contain cardiovascular cell lineage-related genes. A heatmap composed of cardiovascular cell lineage-related genes demonstrated distinct gene expression patterns between the closing DA tissues (Cases 2 and 3) and the patent DA tissue (Case 1) (Figure 4A). The genes *SEMA5A*, *SFRP1*, *NRG1*, *CTNNB1*, *PHACTR4*, and *JAG1* were highly expressed in the ITs of the closing DA tissues. Among these genes, the expression of *PHACTR4* and *JAG1*, which are cardiac neural crest-related genes, was greater in the tunica media of the closing DA tissues than the patent DA tissue. The expression levels of *CFL1*, *TWIST1*, *EDNRB*, *SMO*, and *MAPK1* were greater in the tunica media of the patent DA tissue compared to the closing DA tissues. Similarly, expressions of *SEMA4F*, *NRP1*, *LTBP3*, *EDN3*, and *FGF8* were enriched in the tunica media of the patent DA tissue. *SEMA3G*, *ALX1*, *SOX8*, *ALDH1A2*, and *SEMA7A* were relatively highly expressed in the entire tissue of the patent DA. *WNT8A*, *KLHL12*, *FBXL17*, and *ISL1*, which is a second heart field-related gene, were relatively enriched in the patent DA tissue, and the expression levels of these genes were higher in the IT than in the tunica media.

### 3.6. The Closing or Patent DA Tissue-Specific Gene Expression

Figure 4B presents a Venn diagram that shows probe sets that were upregulated (>8-fold) in the tunica media of the closing DA tissues (Cases 2 and 3) compared to the patent DA tissue (Case 1). Twenty-one overlapped probe sets consisted of 16 genes (Table 3). *APLN*, *CEMIP2*, and *GHRL* are related to vascular development [24,25,26]. There were several genes related to adhesion and protein secretion such as *APLN*, *CD83*, *FLCN*, and *NEDD9* [27,28,29,30]. *GHRL* and *NEDD9* were reported to regulate actin filament organization [31,32]. *APLN* was reported to promote proliferation and migration of vascular SMCs, as well as promote SMC contraction [27]. *NEDD9* is involved in embryonic neural crest cell development and promotes cell migration, cell adhesion, and actin fiber formation [31]. To examine the effect of PGE_1_ administration on the human DAs, we compared gene expressions of the tunica media between shorter-term PGE_1_-treated DAs (Cases 2 and 3) and a longer-term PGE_1_-treated DA (Case 4) using a Venn diagram (Figure S1C, Supplementary Materials). We identified 20 probe sets that overlapped and were enriched in the outer part of a longer-term PGE_1_-treated DA compared to the IT of shorter-term PGE_1_-treated DAs (Table S2, Supplementary Materials). These genes did not belong to the genes in Table 3, suggesting that the genes presented in Table 3 did not seem to have been strongly influenced by PGE_1_ administration.

In the Venn diagram in Figure 4C, 116 probe sets are presented, which were upregulated (>8-fold) in the tunica media of the patent DA tissue (Case 1) compared to the closing DA tissues (Cases 2 and 3). These probe sets contained 52 genes (Table 4). Latent transforming growth factor beta-binding protein 3 (*LTBP3*) was upregulated in the tunica media of the patent DA tissue. *LTBP3* is related to extracellular matrix constituents [33] and second heart field-derived vascular SMCs [34]. Expression of *PRSS55*, identified as an aorta-dominant gene in rodent microarray data [11], was elevated in the patent DA tissue.

### 3.7. Jagged 1 Was Highly Expressed in the Closing DA Tissues

**Figure 5:**
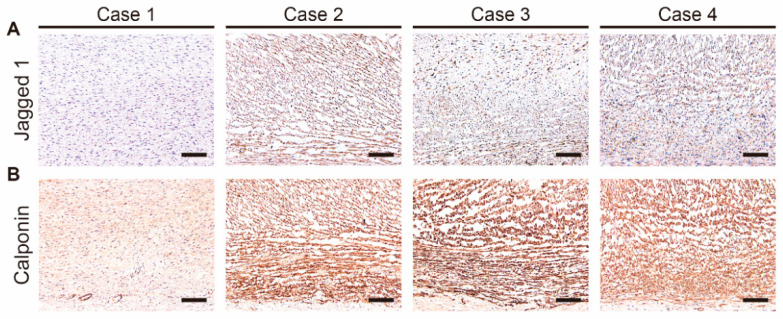
Immunohistochemistry for jagged 1 (**A**) and calponin (**B**) in the human ductus arteriosus tissues from Cases 1–4. A brown color indicates positive immunostaining. Scale bars: 100 µm.

Previous reports using genetically modified mice clearly demonstrated that SMCs of the DA are derived from cardiac neural crest cells, and these cells contribute to SMC differentiation in the DA [35,36]. Since the transcriptome analysis revealed that the neural crest cell-related gene *JAG1* was abundantly expressed in the closing DA tissues compared to the patent DA tissue (Figure 4A), we performed immunohistochemistry to examine protein expression of jagged 1. In agreement with the transcriptome data, jagged 1 was highly expressed in the closing DA tissues (Cases 2–4) (Figure 5A). Calponin is well recognized as a differentiated SMC marker [1], and it was decreased in the DA tissues of *Jag1*-deficient mice [37]. A strong immunoreaction for calponin was observed in the closing DA tissues but was not as strong in the patent DA tissue (Figure 5B).

### 3.8. Transcriptomic Characteristics of the IT and the Tunica Media in the Closing DA Tissues

Lastly, we investigated the difference in gene expression between the IT and the tunica media in the closing DA tissues. IT formation is partly attributed to migration and proliferation of the tunica media-derived SMCs [2,3,4,5,7]. Gene expression analysis indicated that there were different transcriptomic characteristics between the IT and the tunica media in the closing DA tissues (Clusters B1 and B2 in Figure 2). We, thus, compared the expression of genes between the IT and the tunica media of the closing DA tissues (Cases 2 and 3).

**Figure 6:**
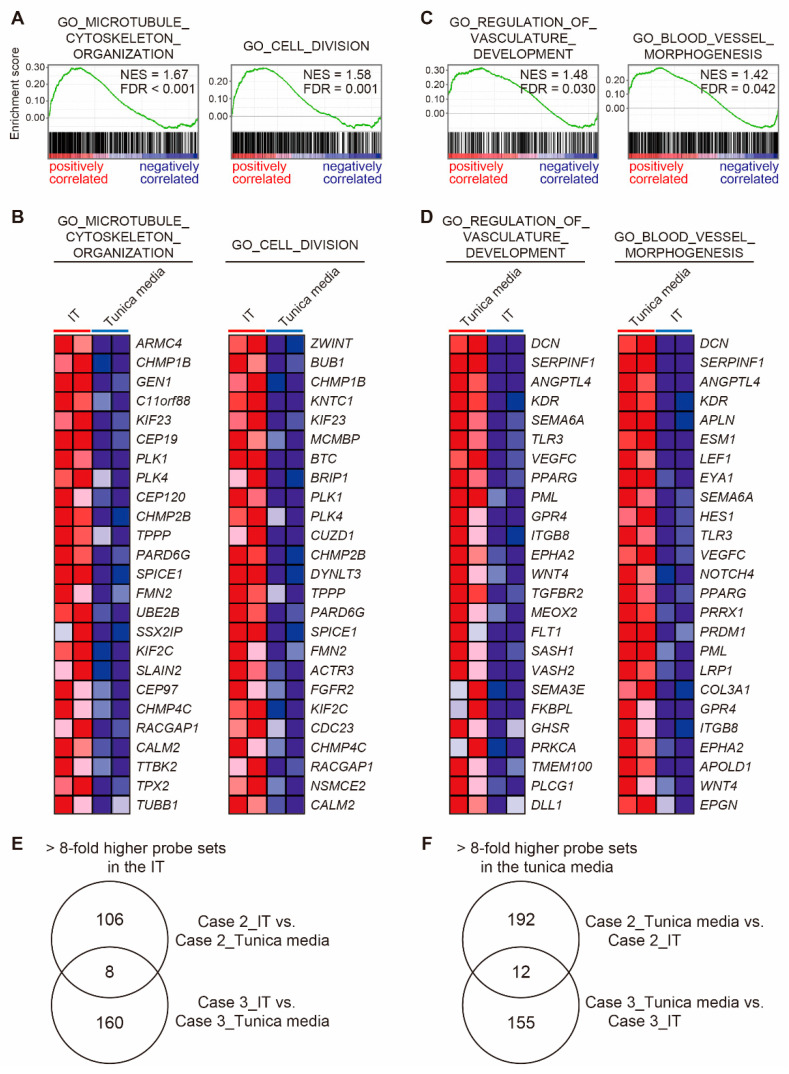
Differential gene expression between the intimal thickening (IT) and the tunica media of the closing human ductus arteriosus (DA) tissues. (**A**) Gene set enrichment analyses (GSEAs) revealed positive correlations between the IT of closing DA tissues and migration- and proliferation–related genes. (**B**) The top 25 genes that comprise the leading edge of the enrichment score in (**A**) are shown as a heatmap. (**C**) GSEAs revealed positive correlations between the tunica media of the closing DA tissues and vascular morphogenesis-related genes. (**D**) The top 25 genes that comprise the leading edge of the enrichment score in (**B**) are shown as a heatmap. (**E**) A Venn diagram shows the number of probe sets that were highly expressed (>8-fold) in the IT compared to the tunica media of closing DA tissues (Cases 2 and 3). (**F**) A Venn diagram shows the numbers of probe sets that were highly expressed (>8-fold) in the tunica media compared to the IT of closing DA tissues (Cases 2 and 3). NES, normalized enrichment score; FDR, false discovery rate.

The GSEAs between the IT and the tunica media, using all gene sets which related to biological processes in the Gene Ontology (GO) (size > 300), were performed. The analyses revealed that the IT was significantly correlated to 89 biological processes (FDR < 0.25, Table 5), and that the tunica media correlated to 81 biological processes (FDR < 0.25, Table 6). The IT of the closing DAs was significantly correlated to more than 10 migration- and proliferation-related gene sets (GO_MICROTUBULE_CYTOSKELETON_ORGANIZATION and GO_CELL_DIVISION, etc.) (Figure 6A,B). Wnt signaling-related gene sets (GO_REGULATION_OF_WNT_SIGNALING_PATHWAY, GO_CANONICAL_WNT_SIGNALING_PATHWAY, and GO_CELL_CELL_SIGNALING_BY_WNT) were also enriched in the IT of the closing DA tissues. The tunica media of the closing DAs was significantly correlated to vascular development-related gene sets (GO_REGULATION_OF VASCULATURE_DEVELOPMENT, GO_BLOOD_VESSEL_MORPHOGENESIS, GO_VASCULAR _DEVELOPMENT, and GO_CIRCULATORY_SYSTEM_DEVELOPMENT) (Figure 6C,D). Five adhesion-related gene sets, including GO_BIOLOGICAL_ADHESION, were enriched in the tunica media compared to the IT in the closing DA tissues.

Figure 6E presents a Venn diagram that shows probe sets that were upregulated (>8-fold) in the IT of closing DA tissues compared to the tunica media of the same DA tissues (Cases 2 and 3). Eight overlapped probe sets consisted of eight genes (Figure 6E and Table 7). *POU4F1*, *FGF1*, and *PROCR* are related to cell division and cell cycle [38,39,40]. *FGF1* is reportedly involved in proliferation and migration of vascular SMCs [39]. A Venn diagram in Figure 6F shows 12 probe sets that were commonly upregulated (>8-fold) in the tunica media of the closing DA tissues compared to the IT of the same DA tissues (Cases 2 and 3), which consisted of eight genes (Figure 6F and Table 8). There were several genes related to muscle structure development such as *BDKRB2*, *MSC*, and *DCN* [41,42,43]. *DCN* is also involved in extracellular constituents and stabilizes collagen and elastic fibers [44,45].

## 4. Discussion

The present study demonstrated that neonatal closing DAs exhibited prominent IT and sparse elastic fiber formation, which are typical human DA characteristics. Postnatal closing DA tissues had abundant expression of cardiac neural crest-related protein jagged 1 and the differentiated smooth muscle marker calponin compared to the patent DA tissue. On the other hand, the patent DA tissue had a distinct morphology (e.g., aorta-like elastic lamellae and a poorly formed IT) and gene profiles, such as second heart field-related genes, compared to the closing DA tissues.

The DA is originally derived from the sixth left aortic artery and has a unique cell-lineage [46]. SMCs of the DA are derived from cardiac neural crest cells [35,47,48] and the DA endothelial cells (ECs) are from second heart field [48,49], while both SMCs and ECs of the adjacent pulmonary artery are derived from second heart field [48,49]. In the ascending aorta, ECs are derived from second heart field, and SMCs of the inner medial layer and outer layer are derived from neural crest cells and second heart field, respectively [47,48]. The heatmap of transcriptome data indicated that these cell lineage-related genes were differentially expressed between the closing DA tissues and the patent DA tissue. Although it was difficult to clearly classify these genes into each lineage due to some overlap, cardiac neural crest-related genes such as *JAG1* were highly expressed in the closing DA tissues. In contrast, second heart field-related genes, such as *ISL1*, were enriched in the patent DA tissue.

The DA has been reported to have differentiated SMCs compared to the adjacent great arteries [1,50]. Slomp et al. demonstrated high levels of calponin expression in the tunica media of the fetal human DA [1]. Similarly, Kim et al. reported the presence of highly differentiated SMCs in the fetal rabbit DA, according to *SM2* expression [50]. These differentiated SMCs have a high contractile apparatus, which makes them compatible with the postnatal potent DA contraction [1,50]. Additionally, several mutant mice with the patent DA had less differentiated SMCs [35,36,37]. Ivey et al. reported that mice lacking *Tfap2β*, which is a neural crest-enriched transcription factor, had decreased expression of calponin on embryonic day 18.5 [36]. Huang et al. utilized mice that harbored a neural crest-restricted deletion of the myocardin gene and demonstrated that decreased SMC contractile proteins were present on embryonic day 16.5 [35]. In addition, SMC-specific *Jag1*-deficient mice had a limited expression of SMC contractile proteins, even at postnatal day 0 [37]. The transcriptome data of the human DA tissues in the present study exhibited decreased protein levels of Jagged 1 and calponin in the patent DA tissue compared to the closing DA tissues. These altered SMC differentiation markers may contribute to postnatal DA patency in humans.

Prematurity and several genetic syndromes are reported to increase the incidence of the patent DA [51], and the patent DA can be classified into three groups, i.e., (1) patent DA in preterm infants, (2) patent DA as a part of a clinical syndrome, and (3) non-syndromic patent DA. This study included only one case with patent DA who had heterotaxy syndrome (polysplenia), which is a major study limitation. Indeed, in the present study, the expression of *Nodal*, which plays a primary role in the determination of left–right asymmetry, was positively correlated to the closing DAs compared to the patent DA with heterotaxy (Figure 3B). Therefore, it was not able to conclude that differentially expressed genes between in the closing DAs and the patent DA were associated with DA patency, but not with heterotaxy.

There are several syndromes (mutated genes) associated with the patent DA, such as Cantú (*ABCC9*), Char (*TFAP2B*), DiGeorge (*TBX1*), Holt-Oram (*TBX5*), and Rubinstein–Taybi (*CREBBP*) syndromes [52,53,54]. In addition to these syndromes, heterotaxy syndrome was reported to have a higher incidence of patent DA [55]. Notch signaling pathways have been reported to play a role in the establishment of left–right asymmetry via regulating *Nodal* expression [56,57]. Mutant for the Notch ligand *Dll1* or double mutants for *Notch1* and *Notch2* exhibited defects in left–right asymmetry [56,57]. *Dll1*-null mutants die at early embryonic days due to severe hemorrhages [58], and it is not yet elucidated whether this *Dll1*-mediated Notch signaling is involved in the pathogenesis of patent DA. It has been reported that combined SMC-specific deletion of *Notch2* and heterozygous deletion of *Notch3* in mice showed the patent DA, but not heterotaxy [59]. In this study, we delineated the low levels of *JAG1*, which is a Notch ligand, in the patent DA with heterotaxy syndrome. Mice with *Jag1*-null mutant are early embryonic lethal due to hemorrhage [60], and SMC-specific *Jag1*-deleted mice are postnatal lethal due to patent DA [37]. These *Jag1* mutants were not reported to exhibit heterotaxy. In humans, there is no obvious relationship between Alagille syndrome (*JAG1*) and patent DA or heterotaxy [61,62]. In mice, phenotypes of patent DA or heterotaxy seem to depend on ligands and isoforms of receptors of Notch signaling. In addition, there are differences in phenotypes caused by genetic mutations between in mice and humans. Analysis of non-syndromic patent DA would provide further insights into molecular mechanisms of closing and patency of the human DA.

Yarboro et al. performed RNA sequencing to determine genes that were differentially expressed in the preterm human DA and aorta at 21 weeks gestation [18], which was a much earlier time point than we used in our study. They found that several previously recognized DA-dominant genes in rodent studies [11,14,15,17] (e.g., *ABCC9*, *PTGER4*, and *TFAP2B*) were also upregulated in the preterm human DA tissues compared to the aorta [18]. Some DA-dominant genes (e.g., *ERG1* and *SFRP1*) in the preterm human DA [18] were upregulated in the closing DAs compared to the patent DA in the present study. In addition, some aorta-dominant genes, including *ALX1* [18], were upregulated in the patent DA compared to the closing DA, which might partly support the aortification phenotype of the patent DA. *Jag1* was reported to be the term DA dominant gene rather than the preterm DA dominant gene in rats [10], suggesting that *Jag1* contributes to normal DA development. In addition to these previously reported genes, the gene profiles in our study potentially provide novel candidate genes (e.g., *APLN* and *LTBP3*) that may contribute to vascular SMC development and function [27,34]. Further study is needed to understand the roles of these genes in DA development.

Mueller et al. performed DNA microarray analysis using postnatal human DAs [19]. Their DA samples were composed of two stent-implanted DAs, one ligamentous DA, and one un-stented open DA [19]. We compared our data to their un-stented open DA dominant genes; however, we could not find obvious overlapped genes among them. One possible reason is that Mueller et al. compared the un-stented open DA on postnatal day 1 to the stented DAs on postnatal days 222 and 239. The stent implantation and time-course of sampling might affect the gene expressions.

This study elucidated the transcriptomic difference between the closing DA tissues and the patent DA tissue in humans. However, one of the limitations in this study pertains to the different durations of PGE_1_ administration. In utero, the DA is dilated by prostaglandin E2 (PGE_2_), which is mainly derived from the placenta [63]. After birth, the loss of the placenta and the increased flow of the lung, which is the major site of PGE catabolism, cause a decline in circulating PGE_2_ [64]. This decline in PGE_2_ contributes to a postnatal DA contraction [64]. We previously reported PGE_2_-induced structural DA remodeling via the prostaglandin receptor EP4 (e.g., IT formation [4,5,22] and attenuation of elastic laminae in the tunica media [6]). In humans, Mitani et al. reported that lipo-PGE_1_ administration increased IT formation in the DA [65]. Gittenberger-de Groot et al. reported that PGE_1_ treatment induced histopathologic changes (e.g., edema) in the human DA [66]. In this study, Case 4 who received the longest PGE_1_ administration, for 98 days, had prominent IT formation and less visible layered elastic fibers. This study demonstrated that the duration of PGE_1_-treatment affected gene expressions such as cell-cycle process-related genes. On the basis of these findings, postnatal PGE_1_ administration was thought to influence not only structural changes but also gene expression in the postnatal DA.

In 1977, Gittenberger-de Groot et al. performed histological analysis of 42 specimens of postnatal human DAs ranging in age from 12 h after premature delivery to 32 years [9]. An abnormal wall structure of the DA was found in all 14 patients that were over 4 months of age, and the most prominent feature was an aberrant distribution of elastic material, such as unfragmented subendothelial elastic lamina [9]. Three of the 14 patients also showed countable elastic laminae in the tunica media, namely, an aortification [9]. The histological finding of patent DA (Case 1) in the present study was consistent with this aortification type, showing aberrant distribution of elastic materials.

As mentioned above, a major limitation of this study is the use of only one sample of the patent DA, which could not represent the whole entity of patent DA. It is difficult to obtain large numbers of samples with a variety of different congenital heart diseases because isolation of the DA is possible only in the case of a limited number of surgical procedures (e.g., aortic arch repair). However, transcriptome comparisons of different types of patent DA tissues would be more informative to elucidate the pathogenesis of the human patent DA.

## 5. Conclusions

Transcriptome analysis using the IT and the tunica media of human DA tissue revealed different gene profiles between the patent DA and the closing DA tissues. Cardiac neural crest-related genes such as *JAG1* were highly expressed in the tunica media and IT of the closing DA tissues compared to the patent DA. Second heart field-related genes, such as *ISL1*, were enriched in the patent DA. The data from this study indicate that patent DA tissue may have different cell lineages from closing DA tissue.

## Figures and Tables

**Table 1:** A summary of the patient profiles for the four cases included in this study.

Case Number	Diagnosis	Gestational Age (Weeks)	Birth Weight (g)	Age at Operation (Days)	Duration of PGE_1_ Administration (Days)	Closing Tendency of the DA
1	Polysplenia, intermediate AVSD, CoA, TAPVC (cardiac type), PDA, IVC interruption (azygos connection)	38	2714	17	0	No
2	DORV (subpulmonary VSD), hypoplastic distal arch, CoA, PFO	41	2948	5	5	Yes
3	HLHS, TR, cor triatriatum, PLSVC	40	3352	24	24	Yes
4	HLHS (MA, AA), PLSVC	37	2654	98	98	Yes

Abbreviations: PGE_1_, prostaglandin E1; DA, ductus arteriosus; AVSD, atrioventricular septal defect; CoA, coarctation of the aorta; TAPVC, total anomalous pulmonary venous connection; PDA, patent ductus arteriosus; IVC, inferior vena cava; DORV, double outlet right ventricle; VSD, ventricular septal defect; PFO, patent foramen ovale; HLHS, hypoplastic left heart syndrome; TR, tricuspid regurgitation; PLSVC, persistent left superior vena cava; MA, mitral atresia; AA, aortic atresia.

**Table 2:** Gene Ontology biological process terms (size > 300) that were significantly upregulated (FDR < 0.25) in the tunica media of the closing human DA tissues (Cases 2 and 3) compared to that of the patent DA tissue (Case 1).

Gene Set Name	Size	NES	FDR *q*-Value	Rank at Max
GO_REGULATION_OF_VASCULATURE_DEVELOPMENT	310	1.61	0.000	6660
GO_BLOOD_VESSEL_MORPHOGENESIS	564	1.61	0.000	6712
GO_GOLGI_VESICLE_TRANSPORT	350	1.61	0.000	8448
GO_REGULATION_OF_HEMOPOIESIS	441	1.60	0.000	6338
GO_NCRNA_PROCESSING	340	1.59	0.000	5889
GO_VIRAL_LIFE_CYCLE	315	1.58	0.000	7537
GO_REGULATION_OF_MAP_KINASE_ACTIVITY	332	1.55	0.000	6500
GO_LEUKOCYTE_CELL_CELL_ADHESION	339	1.54	0.000	5082
GO_NEGATIVE_REGULATION_OF_IMMUNE_SYSTEM_PROCESS	405	1.54	0.000	4982
GO_NEGATIVE_REGULATION_OF_PHOSPHORYLATION	424	1.53	0.000	5671
GO_POSITIVE_REGULATION_OF_CELL_ADHESION	410	1.53	0.000	4942
GO_NEGATIVE_REGULATION_OF_CELL_CYCLE_PROCESS	312	1.53	0.000	5276
GO_NUCLEAR_TRANSPORT	337	1.52	0.000	7197
GO_T_CELL_ACTIVATION	458	1.51	0.000	5293
GO_IN_UTERO_EMBRYONIC_DEVELOPMENT	372	1.47	0.000	6660
GO_REGULATION_OF_INFLAMMATORY_RESPONSE	348	1.47	0.000	5237
GO_PEPTIDYL_LYSINE_MODIFICATION	353	1.46	0.000	8228
GO_REGULATION_OF_PROTEIN_CATABOLIC_PROCESS	372	1.45	0.000	6333
GO_IMMUNE_RESPONSE_REGULATING_SIGNALING_PATHWAY	385	1.40	0.000	7472
GO_CELLULAR_RESPONSE_TO_EXTERNAL_STIMULUS	305	1.40	0.000	7120
GO_REGULATION_OF_CELL_CELL_ADHESION	406	1.39	0.000	5082
GO_RNA_SPLICING	423	1.39	0.000	7328
GO_ACTIVATION_OF_PROTEIN_KINASE_ACTIVITY	321	1.39	0.000	5553
GO_REGULATION_OF_CELLULAR_RESPONSE_TO_STRESS	690	1.39	0.000	6338
GO_RAS_PROTEIN_SIGNAL_TRANSDUCTION	330	1.39	0.000	7040
GO_POSITIVE_REGULATION_OF_PROTEIN_SERINE_THREONINE_KINASE_ACTIVITY	329	1.38	0.000	6543
GO_AMEBOIDAL_TYPE_CELL_MIGRATION	386	1.38	0.000	7624
GO_REGULATION_OF_DNA_BINDING_TRANSCRIPTION_FACTOR_ACTIVITY	415	1.37	0.000	6196
GO_REGULATION_OF_LYMPHOCYTE_ACTIVATION	420	1.35	0.000	5268
GO_RNA_SPLICING_VIA_TRANSESTERIFICATION_REACTIONS	341	1.35	0.000	7328
GO_REGULATION_OF_T_CELL_ACTIVATION	316	1.35	0.000	5256
GO_LYMPHOCYTE_DIFFERENTIATION	354	1.34	0.000	5436
GO_ORGANELLE_FISSION	414	1.34	0.000	4640
GO_POSITIVE_REGULATION_OF_CATABOLIC_PROCESS	430	1.34	0.000	4648
GO_EPITHELIAL_CELL_PROLIFERATION	379	1.33	0.011	6543
GO_REGULATION_OF_PROTEIN_SERINE_THREONINE_KINASE_ACTIVITY	495	1.33	0.010	6256
GO_MRNA_PROCESSING	477	1.33	0.010	7113
GO_EMBRYO_DEVELOPMENT_ENDING_IN_BIRTH_OR_EGG_HATCHING	644	1.32	0.010	6259
GO_NEGATIVE_REGULATION_OF_INTRACELLULAR_SIGNAL_TRANSDUCTION	475	1.32	0.009	7981
GO_PROTEIN_POLYUBIQUITINATION	327	1.31	0.014	6212
GO_RIBONUCLEOPROTEIN_COMPLEX_BIOGENESIS	405	1.31	0.013	6510
GO_MAINTENANCE_OF_LOCATION	305	1.31	0.013	5837
GO_LEUKOCYTE_DIFFERENTIATION	514	1.31	0.013	7110
GO_POSITIVE_REGULATION_OF_CELL_CYCLE	357	1.31	0.013	5009
GO_POSTTRANSCRIPTIONAL_REGULATION_OF_GENE_EXPRESSION	554	1.29	0.012	6212
GO_RESPONSE_TO_OXYGEN_LEVELS	370	1.29	0.012	6459
GO_POSITIVE_REGULATION_OF_ESTABLISHMENT_OF_PROTEIN_LOCALIZATION	371	1.28	0.012	6982
GO_REGULATION_OF_METAL_ION_TRANSPORT	363	1.28	0.012	4661
GO_PROTEASOMAL_PROTEIN_CATABOLIC_PROCESS	455	1.28	0.011	5596
GO_NEGATIVE_REGULATION_OF_PHOSPHORUS_METABOLIC_PROCESS	530	1.26	0.011	5671
GO_POSITIVE_REGULATION_OF_GTPASE_ACTIVITY	375	1.26	0.011	7037
GO_REGULATION_OF_GTPASE_ACTIVITY	447	1.26	0.011	7095
GO_POSITIVE_REGULATION_OF_RESPONSE_TO_EXTERNAL_STIMULUS	496	1.25	0.010	6529
GO_COVALENT_CHROMATIN_MODIFICATION	436	1.25	0.010	6473
GO_NEURON_DEATH	338	1.25	0.010	7040
GO_POSITIVE_REGULATION_OF_PROTEOLYSIS	340	1.24	0.010	6359
GO_POSITIVE_REGULATION_OF_CELLULAR_PROTEIN_LOCALIZATION	307	1.24	0.010	6982
GO_POSITIVE_REGULATION_OF_CYTOKINE_PRODUCTION	432	1.24	0.010	5082
GO_PROCESS_UTILIZING_AUTOPHAGIC_MECHANISM	495	1.23	0.009	5327
GO_VESICLE_ORGANIZATION	315	1.23	0.009	7253
GO_POSITIVE_REGULATION_OF_CELL_ACTIVATION	324	1.23	0.009	7094
GO_POST_TRANSLATIONAL_PROTEIN_MODIFICATION	352	1.23	0.009	7885
GO_LEUKOCYTE_MIGRATION	428	1.22	0.009	7094
GO_ESTABLISHMENT_OF_ORGANELLE_LOCALIZATION	397	1.22	0.009	6914
GO_CANONICAL_WNT_SIGNALING_PATHWAY	315	1.20	0.009	5210
GO_REGULATION_OF_CELLULAR_AMIDE_METABOLIC_PROCESS	385	1.19	0.008	6376
GO_REGULATION_OF_AUTOPHAGY	327	1.18	0.008	4699
GO_REGULATION_OF_CHROMOSOME_ORGANIZATION	321	1.17	0.014	6695
GO_REGULATION_OF_SMALL_GTPASE_MEDIATED_SIGNAL_TRANSDUCTION	312	1.16	0.024	7040
GO_REPRODUCTIVE_SYSTEM_DEVELOPMENT	428	1.16	0.026	3453
GO_REGULATION_OF_SUPRAMOLECULAR_FIBER_ORGANIZATION	339	1.15	0.028	6891
GO_POSITIVE_REGULATION_OF_DEFENSE_RESPONSE	360	1.14	0.048	5237
GO_POSITIVE_REGULATION_OF_NERVOUS_SYSTEM_DEVELOPMENT	513	1.14	0.047	4697
GO_REGULATION_OF_APOPTOTIC_SIGNALING_PATHWAY	383	1.13	0.047	5796
GO_MYELOID_CELL_DIFFERENTIATION	375	1.12	0.061	6268
GO_RESPONSE_TO_VIRUS	315	1.12	0.060	5563
GO_ACTIN_FILAMENT_ORGANIZATION	400	1.11	0.077	6891
GO_RESPONSE_TO_MOLECULE_OF_BACTERIAL_ORIGIN	326	1.10	0.097	6178
GO_REGULATION_OF_PROTEIN_CONTAINING_COMPLEX_ASSEMBLY	408	1.10	0.098	6574
GO_OSSIFICATION	378	1.10	0.106	6790
GO_POSITIVE_REGULATION_OF_SECRETION	358	1.09	0.109	6953
GO_EPITHELIAL_TUBE_MORPHOGENESIS	326	1.09	0.126	6477
GO_ACTIVATION_OF_IMMUNE_RESPONSE	433	1.09	0.138	7472
GO_CELL_SUBSTRATE_ADHESION	342	1.08	0.172	8285
GO_REGULATION_OF_BINDING	349	1.08	0.178	6143
GO_REGULATION_OF_DEVELOPMENTAL_GROWTH	324	1.08	0.178	4701
GO_CELLULAR_RESPONSE_TO_CHEMICAL_STRESS	329	1.07	0.229	4800

Abbreviations: NES, normalized enrichment score; FDR, false discovery rate.

**Table 3:** Sixteen genes that overlapped and were enriched (>8-fold) in the tunica media of the closing DA tissues (Cases 2 and 3) compared to that of the patent DA tissue (Case 1).

Gene Name	Description	Fold Change	
		Case 2 vs. Case 1	Case 3 vs. Case 1
*CD83*	*CD83* molecule	29.9	29.9
*AP1S3*	adaptor-related protein complex 1 subunit sigma 3	26.5	18.0
*GSTT1*	glutathione *S*-transferase theta 1	21.6	10.7
*BCL2L13*	*BCL2*-like 13	12.9	13.1
*NEDD9*	neural precursor cell expressed, developmentally downregulated 9	12.1	13.6
*HLA-DMA*	major histocompatibility complex, class II, DM alpha	9.1	16.3
*GHRL*	ghrelin and obestatin prepropeptide	13.1	12.2
*FLCN*	folliculin	12.2	9.1
*TCF7*	transcription factor 7, T-cell-specific	11.0	9.0
*ELOVL5*	*ELOVL* fatty-acid elongase 5	9.0	9.9
*APLN*	apelin	9.2	9.6
*MAFF*	*MAF* bZIP transcription factor F	9.5	8.8
*AURKAPS1*	aurora kinase A pseudogene 1	9.7	8.4
*MIS12*	*MIS12* kinetochore complex component	9.5	8.3
*CEMIP2*	cell migration inducing hyaluronidase 2	8.4	9.1
*GMCL1*	germ cell-less 1, spermatogenesis associated	8.5	8.5

**Table 4:** Fifty-two genes that overlapped and were enriched (>8-fold) in the tunica media of the patent DA tissue (Case 1) compared to that of the closing DA tissues (Cases 2 and 3).

Gene Name	Description	Fold Change	
		Case 1 vs. Case 2	Case 1 vs. Case 3
*MYH16*	myosin heavy chain 16 pseudogene	70.1	68.9
*PRDM12*	PR/SET domain 12	69.8	68.5
*CXXC4*	*CXXC* finger protein 4	62.8	61.5
*VENTXP1*	*VENT* homeobox pseudogene 1	60.4	57.3
*MKRN3*	makorin ring finger protein 3	70.9	43.7
*CLEC3A*	C-type lectin domain family 3 member A	26.1	251.3
*TEX43*	testis expressed 43	51.3	26.7
*SCN11A*	sodium channel, voltage-gated, type XI, alpha subunit 11	38.0	26.5
*CYP3A43*	cytochrome P450 family 3 subfamily A member 43	24.5	22.3
*MROH2A*	maestro heat-like repeat family member 2A	21.1	21.4
*MUC12*	mucin 12, cell-surface-associated	30.5	15.3
*MS4A6A*	membrane-spanning 4-domains subfamily A member 6A	46.8	11.7
*RBFOX3*	RNA-binding fox-1 homolog 3	15.5	20.4
*SLC2A1*	solute carrier family 2 member 1	30.4	10.7
*CFAP299*	cilia- and flagella-associated protein 299	15.4	15.8
*PSG5*	pregnancy-specific beta-1-glycoprotein 5	16.3	14.6
*LTBP3*	latent transforming growth factor beta-binding protein 3	13.7	16.9
*ZFP57*	zinc finger protein 57	21.8	11.0
*MFSD4*	major facilitator superfamily domain-containing 4A	13.8	13.8
*HOXA11*	homeobox A11	10.2	19.1
*ALLC*	allantoicase	24.8	8.8
*SLC6A14*	solute carrier family 6 member 14	13.1	12.1
*SLC44A4*	solute carrier family 44 member 4	12.7	11.7
*MAS1*	*MAS1* proto-oncogene, G-protein-coupled receptor	12.2	12.2
*CARD18*	caspase recruitment domain family member 18	12.9	11.5
*LCE1C*	late cornified envelope protein 1C	12.2	10.6
*PAMR1*	peptidase domain-containing associated with muscle regeneration 1	8.1	18.7
*PRSS55*	serine protease 55	11.4	10.5
*RUNDC3B*	*RUN* domain-containing 3B	10.0	11.8
*LINC00114*	long intergenic non-protein-coding RNA 114	8.9	13.8
*TFAP4*	transcription factor AP-4	11.2	10.1
*PLIN2*	perilipin 2	9.8	11.1
*CCR2*	C–C motif chemokine receptor 2	11.7	9.3
*CDKN2B-AS*	*CDKN2B* antisense RNA 1	10.0	9.8
*MYO7A*	myosin VIIA	10.4	9.3
*PDILT*	protein disulfide isomerase like, testis expressed	9.6	9.6
*SPA17*	sperm autoantigenic protein 17	10.8	8.5
*SLITRK2*	*SLIT* and *NTRK*-like family member 2	9.5	9.5
*SLC9B1*	solute carrier family 9 member B1	9.3	9.7
*PANX2*	pannexin 2	11.4	8.0
*PLPPR1*	phospholipid phosphatase-related 1	10.1	8.8
*ASTE1*	asteroid homolog 1	9.2	9.4
*MUC16*	mucin 16, cell-surface-associated	9.5	8.7
*OR51B2*	olfactory receptor family 51 subfamily B member 2	9.2	8.6
*ARHGAP36*	Rho GTPase-activating protein 36	8.8	8.7
*KRTAP4-8*	keratin-associated protein 4-8	8.8	8.5
*METTL21CP1*	methyltransferase-like 21E, pseudogene	9.1	8.3
*BPIFB6*	BPI fold-containing family B member 6	8.9	8.4
*HABP2*	hyaluronan-binding protein 2	9.0	8.2
*DUSP13*	dual-specificity phosphatase 13	8.9	8.2
*CXorf51A*	chromosome X open reading frame 51A	8.2	8.0
*MTM1*	myotubularin 1	8.2	8.1

**Table 5:** Gene Ontology biological process terms (size > 300) that were significantly upregulated (FDR < 0.25) in the intimal thickening compared to the tunica media of the closing DA tissues (Cases 2 and 3).

Gene Set Name	Size	NES	FDR *q*-Value	Rank at Max
GO_MICROTUBULE_CYTOSKELETON_ORGANIZATION	526	1.67	0.000	6797
GO_MICROTUBULE_BASED_PROCESS	757	1.59	0.000	6797
GO_CELL_DIVISION	549	1.58	0.000	6799
GO_PROTEIN_POLYUBIQUITINATION	327	1.56	0.000	7778
GO_MITOTIC_CELL_CYCLE	954	1.54	0.000	6055
GO_MODIFICATION_DEPENDENT_MACROMOLECULE_CATABOLIC_PROCESS	604	1.51	0.000	6933
GO_NEGATIVE_REGULATION_OF_CELL_CYCLE	566	1.51	0.000	6548
GO_NEGATIVE_REGULATION_OF_CELL_CYCLE_PROCESS	312	1.50	0.001	6055
GO_MRNA_PROCESSING	477	1.50	0.001	6995
GO_RNA_SPLICING_VIA_TRANSESTERIFICATION_REACTIONS	341	1.48	0.001	6758
GO_ESTABLISHMENT_OF_ORGANELLE_LOCALIZATION	397	1.47	0.001	6823
GO_REGULATION_OF_MRNA_METABOLIC_PROCESS	326	1.47	0.001	9158
GO_ORGANELLE_LOCALIZATION	602	1.46	0.001	6823
GO_RNA_SPLICING	423	1.45	0.002	6007
GO_CELL_CYCLE	1681	1.44	0.002	6059
GO_ORGANELLE_FISSION	414	1.44	0.002	5836
GO_CELL_CYCLE_PROCESS	1251	1.43	0.002	6470
GO_REGULATION_OF_MITOTIC_CELL_CYCLE	600	1.43	0.002	6548
GO_MUSCLE_TISSUE_DEVELOPMENT	368	1.42	0.003	4069
GO_CELLULAR_PROTEIN_CATABOLIC_PROCESS	733	1.41	0.005	6933
GO_REGULATION_OF_CELL_CYCLE_PROCESS	706	1.41	0.005	6450
GO_CELL_CYCLE_PHASE_TRANSITION	578	1.40	0.005	6055
GO_REGULATION_OF_CELL_CYCLE	1110	1.40	0.004	6548
GO_MICROTUBULE_BASED_MOVEMENT	321	1.37	0.007	5942
GO_PROTEIN_MODIFICATION_BY_SMALL_PROTEIN_CONJUGATION_OR_REMOVAL	1033	1.36	0.008	6585
GO_PROTEIN_CATABOLIC_PROCESS	876	1.36	0.008	7607
GO_PROTEASOMAL_PROTEIN_CATABOLIC_PROCESS	455	1.34	0.011	7536
GO_POST_TRANSLATIONAL_PROTEIN_MODIFICATION	352	1.33	0.013	4520
GO_VESICLE_ORGANIZATION	315	1.33	0.013	6530
GO_PROTEIN_MODIFICATION_BY_SMALL_PROTEIN_CONJUGATION	864	1.32	0.014	7634
GO_PROTEIN_CONTAINING_COMPLEX_DISASSEMBLY	310	1.32	0.014	6434
GO_CHROMOSOME_ORGANIZATION	1059	1.32	0.015	6004
GO_RNA_PROCESSING	1149	1.31	0.016	7021
GO_REGULATION_OF_CHROMOSOME_ORGANIZATION	321	1.29	0.024	7299
GO_REGULATION_OF_CELL_CYCLE_PHASE_TRANSITION	424	1.29	0.024	6055
GO_MRNA_METABOLIC_PROCESS	789	1.28	0.026	7021
GO_REGULATION_OF_INTRACELLULAR_TRANSPORT	325	1.28	0.026	6449
GO_MUSCLE_SYSTEM_PROCESS	423	1.28	0.028	3948
GO_CELLULAR_MACROMOLECULE_CATABOLIC_PROCESS	1100	1.26	0.034	7104
GO_REGULATION_OF_AUTOPHAGY	327	1.25	0.041	6901
GO_CELLULAR_PROTEIN_CONTAINING_COMPLEX_ASSEMBLY	909	1.25	0.041	6799
GO_REGULATION_OF_WNT_SIGNALING_PATHWAY	347	1.25	0.042	3438
GO_RIBONUCLEOPROTEIN_COMPLEX_BIOGENESIS	405	1.24	0.042	8496
GO_PROCESS_UTILIZING_AUTOPHAGIC_MECHANISM	495	1.23	0.049	6585
GO_ANATOMICAL_STRUCTURE_HOMEOSTASIS	426	1.23	0.048	6332
GO_ORGANOPHOSPHATE_BIOSYNTHETIC_PROCESS	526	1.23	0.049	3613
GO_REGULATION_OF_CELLULAR_CATABOLIC_PROCESS	812	1.23	0.051	6913
GO_PROTEIN_CONTAINING_COMPLEX_SUBUNIT_ORGANIZATION	1716	1.23	0.052	5624
GO_REGULATION_OF_SYSTEM_PROCESS	571	1.22	0.057	3069
GO_GLYCEROPHOSPHOLIPID_METABOLIC_PROCESS	325	1.22	0.055	2842
GO_ORGANELLE_ASSEMBLY	780	1.22	0.055	5055
GO_MUSCLE_CONTRACTION	339	1.20	0.070	3948
GO_REGULATION_OF_CELLULAR_LOCALIZATION	939	1.20	0.073	6537
GO_CYTOSKELETON_ORGANIZATION	1278	1.20	0.072	5064
GO_REGULATION_OF_CATABOLIC_PROCESS	960	1.20	0.075	7638
GO_MACROMOLECULE_CATABOLIC_PROCESS	1319	1.19	0.077	7614
GO_ORGANONITROGEN_COMPOUND_CATABOLIC_PROCESS	1233	1.19	0.080	6337
GO_CANONICAL_WNT_SIGNALING_PATHWAY	315	1.18	0.084	3438
GO_DIVALENT_INORGANIC_CATION_TRANSPORT	443	1.18	0.087	2865
GO_MUSCLE_STRUCTURE_DEVELOPMENT	606	1.18	0.094	4139
GO_POSITIVE_REGULATION_OF_ESTABLISHMENT_OF_PROTEIN_LOCALIZATION	371	1.18	0.092	5436
GO_DNA_METABOLIC_PROCESS	822	1.18	0.091	7381
GO_CELL_CELL_SIGNALING_BY_WNT	488	1.17	0.102	3490
GO_INTRACELLULAR_TRANSPORT	1599	1.16	0.108	6629
GO_MUSCLE_ORGAN_DEVELOPMENT	360	1.16	0.108	4772
GO_REGULATION_OF_PEPTIDE_TRANSPORT	641	1.16	0.109	5020
GO_SECOND_MESSENGER_MEDIATED_SIGNALING	412	1.15	0.125	3308
GO_PEPTIDE_SECRETION	495	1.15	0.125	3880
GO_REGULATION_OF_CYTOSKELETON_ORGANIZATION	513	1.15	0.128	3944
GO_SIGNAL_RELEASE	577	1.14	0.130	3898
GO_DNA_REPAIR	480	1.14	0.132	6578
GO_MUSCLE_CELL_DIFFERENTIATION	338	1.14	0.131	4061
GO_NEGATIVE_REGULATION_OF_PROTEIN_MODIFICATION_PROCESS	565	1.14	0.132	5343
GO_NEGATIVE_REGULATION_OF_PHOSPHORYLATION	424	1.14	0.138	5364
GO_MITOCHONDRION_ORGANIZATION	459	1.14	0.138	7936
GO_NEGATIVE_REGULATION_OF_PHOSPHORUS_METABOLIC_PROCESS	530	1.13	0.144	5394
GO_HORMONE_TRANSPORT	309	1.12	0.165	3880
GO_REGULATION_OF_PROTEIN_CATABOLIC_PROCESS	372	1.12	0.176	7675
GO_REGULATION_OF_PROTEIN_LOCALIZATION	905	1.11	0.184	6577
GO_RIBOSE_PHOSPHATE_METABOLIC_PROCESS	373	1.11	0.183	4526
GO_OSSIFICATION	378	1.11	0.180	5020
GO_REGULATION_OF_DNA_METABOLIC_PROCESS	314	1.11	0.192	7720
GO_NUCLEOBASE_CONTAINING_SMALL_MOLECULE_METABOLIC_PROCESS	545	1.11	0.196	3618
GO_PHOSPHOLIPID_METABOLIC_PROCESS	420	1.10	0.215	5395
GO_REGULATION_OF_ORGANELLE_ORGANIZATION	1209	1.10	0.220	5212
GO_ORGANOPHOSPHATE_METABOLIC_PROCESS	950	1.09	0.238	3618
GO_REGULATION_OF_CELLULAR_RESPONSE_TO_STRESS	690	1.09	0.235	6943
GO_CELLULAR_RESPONSE_TO_DNA_DAMAGE_STIMULUS	761	1.09	0.246	6578
GO_GLYCEROLIPID_METABOLIC_PROCESS	409	1.09	0.246	5591

Abbreviations: NES, normalized enrichment score; FDR, false discovery rate.

**Table 6:** Gene Ontology biological process terms (size > 300) that were significantly upregulated (FDR < 0.25) in the tunica media compared to the intimal thickening of the closing DA tissues (Cases 2 and 3).

Gene Set Name	Size	NES	FDR *q*-Value	Rank at Max
GO_EXTRACELLULAR_STRUCTURE_ORGANIZATION	376	1.67	0.002	5349
GO_SKELETAL_SYSTEM_DEVELOPMENT	494	1.55	0.012	5310
GO_REGULATION_OF_VASCULATURE_DEVELOPMENT	310	1.49	0.030	7236
GO_EMBRYONIC_ORGAN_DEVELOPMENT	443	1.49	0.023	6270
GO_PATTERN_SPECIFICATION_PROCESS	442	1.48	0.021	6393
GO_INFLAMMATORY_RESPONSE	706	1.45	0.029	7279
GO_TAXIS	612	1.45	0.027	7278
GO_NEGATIVE_REGULATION_OF_CELL_DEVELOPMENT	311	1.44	0.030	6296
GO_BLOOD_VESSEL_MORPHOGENESIS	564	1.43	0.030	7164
GO_NEGATIVE_REGULATION_OF_CELL_DIFFERENTIATION	668	1.42	0.032	6377
GO_POSITIVE_REGULATION_OF_NERVOUS_SYSTEM_DEVELOPMENT	513	1.42	0.036	6270
GO_REGIONALIZATION	347	1.40	0.043	6393
GO_VASCULATURE_DEVELOPMENT	676	1.39	0.050	6711
GO_EMBRYONIC_MORPHOGENESIS	578	1.39	0.050	5668
GO_POSITIVE_REGULATION_OF_CELL_DEVELOPMENT	528	1.38	0.052	5938
GO_REGULATION_OF_NERVOUS_SYSTEM_DEVELOPMENT	888	1.37	0.058	6409
GO_BIOLOGICAL_ADHESION	1379	1.37	0.062	7362
GO_EPITHELIAL_TUBE_MORPHOGENESIS	326	1.36	0.069	6392
GO_TUBE_MORPHOGENESIS	808	1.35	0.075	6726
GO_REGULATION_OF_NEURON_DIFFERENTIATION	631	1.35	0.073	4480
GO_POSITIVE_REGULATION_OF_DEVELOPMENTAL_PROCESS	1298	1.35	0.071	6726
GO_REGULATION_OF_INFLAMMATORY_RESPONSE	348	1.34	0.074	6671
GO_POSITIVE_REGULATION_OF_CELL_DIFFERENTIATION	939	1.34	0.072	6708
GO_AMEBOIDAL_TYPE_CELL_MIGRATION	386	1.34	0.070	3718
GO_POSITIVE_REGULATION_OF_MULTICELLULAR_ORGANISMAL_PROCESS	1662	1.34	0.067	6427
GO_REGULATION_OF_CELL_ADHESION	682	1.34	0.071	7361
GO_CELL_MORPHOGENESIS_INVOLVED_IN_NEURON_DIFFERENTIATION	585	1.33	0.075	6775
GO_NEGATIVE_REGULATION_OF_DEVELOPMENTAL_PROCESS	905	1.33	0.075	6377
GO_AXON_DEVELOPMENT	512	1.32	0.079	7161
GO_TUBE_DEVELOPMENT	998	1.32	0.080	6726
GO_NEUROGENESIS	1571	1.32	0.079	6708
GO_REGULATION_OF_ANATOMICAL_STRUCTURE_MORPHOGENESIS	1032	1.32	0.079	6334
GO_REGULATION_OF_CELL_DIFFERENTIATION	1729	1.31	0.086	6377
GO_REGULATION_OF_T_CELL_ACTIVATION	316	1.31	0.084	5912
GO_POSITIVE_REGULATION_OF_CELL_ADHESION	410	1.31	0.083	6893
GO_REGULATION_OF_CELL_DEVELOPMENT	904	1.31	0.086	6400
GO_REGULATION_OF_CELL_MORPHOGENESIS	474	1.30	0.102	6663
GO_REGULATION_OF_CELL_CELL_ADHESION	406	1.29	0.105	6811
GO_ANATOMICAL_STRUCTURE_FORMATION_INVOLVED_IN_MORPHOGENESIS	1044	1.29	0.106	7374
GO_EPITHELIAL_CELL_PROLIFERATION	379	1.28	0.119	6725
GO_POSITIVE_REGULATION_OF_NEURON_DIFFERENTIATION	356	1.28	0.120	6270
GO_REGULATION_OF_PEPTIDASE_ACTIVITY	419	1.28	0.118	6411
GO_LEUKOCYTE_CELL_CELL_ADHESION	339	1.28	0.116	6889
GO_CELL_CELL_ADHESION	826	1.28	0.119	7362
GO_CELL_MORPHOGENESIS	996	1.27	0.118	6775
GO_MORPHOGENESIS_OF_AN_EPITHELIUM	539	1.27	0.125	6433
GO_CIRCULATORY_SYSTEM_DEVELOPMENT	1018	1.27	0.127	6433
GO_POSITIVE_REGULATION_OF_HYDROLASE_ACTIVITY	719	1.26	0.132	6562
GO_CELLULAR_PROCESS_INVOLVED_IN_REPRODUCTION_IN_MULTICELLULAR_ORGANISM	330	1.26	0.131	6092
GO_GLAND_DEVELOPMENT	436	1.26	0.143	6386
GO_NEGATIVE_REGULATION_OF_MULTICELLULAR_ORGANISMAL_PROCESS	1145	1.25	0.147	6749
GO_REGULATION_OF_CELLULAR_COMPONENT_SIZE	360	1.25	0.152	3939
GO_SENSORY_ORGAN_DEVELOPMENT	560	1.25	0.155	5533
GO_NEURON_DIFFERENTIATION	1327	1.25	0.155	6400
GO_REPRODUCTIVE_SYSTEM_DEVELOPMENT	428	1.25	0.154	4925
GO_CELL_PART_MORPHOGENESIS	680	1.24	0.155	6775
GO_NEURON_DEVELOPMENT	1080	1.24	0.155	6705
GO_T_CELL_ACTIVATION	458	1.24	0.154	7224
GO_POSITIVE_REGULATION_OF_CELL_PROJECTION_ORGANIZATION	366	1.24	0.169	6270
GO_ANIMAL_ORGAN_MORPHOGENESIS	1048	1.24	0.168	6373
GO_ORGANIC_ANION_TRANSPORT	491	1.22	0.168	6889
GO_CELL_MORPHOGENESIS_INVOLVED_IN_DIFFERENTIATION	728	1.22	0.168	6775
GO_ANION_TRANSMEMBRANE_TRANSPORT	303	1.22	0.191	8857
GO_ALCOHOL_METABOLIC_PROCESS	362	1.22	0.192	7136
GO_G_PROTEIN_COUPLED_RECEPTOR_SIGNALING_PATHWAY	1235	1.22	0.195	9098
GO_EMBRYO_DEVELOPMENT	1018	1.22	0.194	6690
GO_ANION_TRANSPORT	628	1.21	0.204	6889
GO_REGULATION_OF_NEURON_PROJECTION_DEVELOPMENT	486	1.21	0.202	4480
GO_REGULATION_OF_CELL_PROJECTION_ORGANIZATION	652	1.21	0.203	6334
GO_LIPID_CATABOLIC_PROCESS	327	1.21	0.203	6013
GO_UROGENITAL_SYSTEM_DEVELOPMENT	325	1.20	0.233	6411
GO_TISSUE_MORPHOGENESIS	639	1.20	0.233	6433
GO_REGULATION_OF_HEMOPOIESIS	441	1.20	0.236	7164
GO_REGULATION_OF_HYDROLASE_ACTIVITY	1205	1.20	0.236	6564
GO_REGULATION_OF_IMMUNE_SYSTEM_PROCESS	1387	1.20	0.235	7164
GO_LYMPHOCYTE_DIFFERENTIATION	354	1.19	0.243	8193
GO_EPITHELIUM_DEVELOPMENT	1261	1.19	0.240	6313
GO_HEAD_DEVELOPMENT	765	1.19	0.238	7239
GO_ENDOCYTOSIS	541	1.19	0.238	5944
GO_TRANSMEMBRANE_RECEPTOR_PROTEIN_TYROSINE_KINASE_SIGNALING_PATHWAY	695	1.19	0.245	7173
GO_REGULATION_OF_CELL_ACTIVATION	538	1.19	0.247	7342

Abbreviations: NES, normalized enrichment score; FDR, false discovery rate.

**Table 7:** Eight genes that overlapped and were enriched (>8-fold) in the intimal thickening (IT) compared to the tunica media of the closing DA tissues (Cases 2 and 3).

Gene Name	Description	Fold Change IT vs. the Tunica Media	
		Case 2	Case 3
*POU4F1*	*POU* class 4 homeobox 1	22.1	16.4
*BMX*	*BMX* non-receptor tyrosine kinase	10.6	29.2
*FGF1*	fibroblast growth factor 1	15.9	9.0
*MPZL2*	myelin protein zero-like 2	18.8	10.2
*FMO3*	flavin-containing dimethylaniline monooxygenase 3	12.8	10.0
*PROCR*	protein C receptor	13.0	8.6
*DSP*	desmoplakin	9.3	11.7
*NR1I2*	nuclear receptor subfamily 1 group I member 2	9.7	10.7

**Table 8:** Eight genes that overlapped and were enriched (>8-fold) in the tunica media compared to the intimal thickening (IT) of the closing DA tissues (Cases 2 and 3).

Gene Name	Description	Fold Change the Tunica Media vs. IT	
		Case 2	Case 3
*GAS7*	growth arrest-specific 7	52.4	9.8
*H19*	*H19* imprinted maternally expressed transcript	14.2	18.5
*BTNL9*	butyrophilin-like 9	8.4	17.9
*SELENOP*	selenoprotein P	14.8	13.4
*BDKRB2*	bradykinin receptor B2	11.3	11.6
*CHRDL1*	chordin-like 1	9.2	23.6
*MSC*	musculin	11.7	8.4
*DCN*	decorin	9.0	9.4

## Data Availability

The data presented in this study are available within the article.

## Supplementary Materials

The following are available online at https://www.mdpi.com/article/10.3390/jcdd8040045/s1: Figure S1. Differential gene expression between the longer-term PGE_1_-treated human ductus arteriosus (DA) tissue (Case 4) and the shorter-term PGE_1_-treated DA tissues (Cases 2 and 3); Table S1. Gene Ontology biological process terms (size > 300) that were significantly upregulated (FDR < 0.25) in the outer part of long-term PGE_1_-treated human ductus arteriosus (DA) tissue (Case 4) compared to the tunica media of short-term PGE_1_-treated DA tissues (Case 2 and Case 3); Table S2. Twenty genes that overlapped and were enriched (>8-fold) in the outer part of a longer-term PGE_1_-treated DA tissue (Case 4) compared to the IT of shorter-term PGE_1_-treated DA tissues (Cases 2 and 3).

## References

[1] Slomp J., de Groot A.C.G., Glukhova M.A., van Munsteren J.C., Kockx M.M., Schwartz S.M., Kote-liansky V.E. (1997). Differentiation, dedifferentiation, and apoptosis of smooth muscle cells during the development of the human ductus arteriosus. Arterioscler. Thromb. Vasc. Biol..

[2] Yokoyama U., Minamisawa S., Ishikawa Y. (2010). Regulation of vascular tone and remodeling of the ductus arteriosus. J. Smooth Muscle Res..

[3] Yokoyama U., Minamisawa S., Katayama A., Tang T., Suzuki S., Iwatsubo K., Iwasaki S., Kurotani R., Okumura S., Sato M. (2010). Differential Regulation of Vascular Tone and Remodeling via Stimulation of Type 2 and Type 6 Adenylyl Cyclases in the Ductus Arteriosus. Circ. Res..

[4] Yokoyama U., Minamisawa S., Quan H., Akaike T., Suzuki S., Jin M., Jiao Q., Watanabe M., Otsu K., Iwasaki S. (2008). Prostaglandin e2-activated epac promotes neointimal formation of the rat ductus arteriosus by a process distinct from that of camp-dependent protein kinase A. J. Biol. Chem..

[5] Yokoyama U., Minamisawa S., Quan H., Ghatak S., Akaike T., Segi-Nishida E., Iwasaki S., Iwamoto M., Misra S., Tamura K. (2006). Chronic activation of the prostaglandin receptor ep4 promotes hyaluronan-mediated neointimal formation in the ductus arteriosus. J. Clin. Investig..

[6] Yokoyama U., Minamisawa S., Shioda A., Ishiwata R., Jin M.H., Masuda M., Asou T., Sugimoto Y., Aoki H., Nakamura T. (2014). Prostaglandin E2 Inhibits Elastogenesis in the Ductus Arteriosus via EP4 Signaling. Circulation.

[7] Yokoyama U. (2015). Prostaglandin E-mediated molecular mechanisms driving remodeling of the ductus arteriosus. Pediatr. Int..

[8] Forsey J.T., Elmasry O.A., Martin R.P. (2009). Patent arterial duct. Orphanet J. Rare Dis..

[9] De Groot A.C.G. (1977). Persistent ductus arteriosus: Most probably a primary congenital malformation. Br. Heart J..

[10] Yokoyama U., Sato Y., Akaike T., Ishida S., Sawada J., Nagao T., Quan H., Jin M., Iwamoto M., Yokota S. (2007). Maternal vitamin A alters gene profiles and structural maturation of the rat ductus arteriosus. Physiol. Genom..

[11] Jin M.H., Yokoyama U., Sato Y., Shioda A., Jiao Q., Ishikawa Y., Minamisawa S. (2011). DNA microarray profiling identified a new role of growth hormone in vascular remodeling of rat ductus arteriosus. J. Physiol. Sci..

[12] Liu N.M., Yokota T., Maekawa S., Lu P., Zheng Y.W., Taniguchi H., Yokoyama U., Kato T., Minamisawa S. (2013). Transcription profiles of endothelial cells in the rat ductus arteriosus during a perinatal period. PLoS ONE.

[13] Goyal R., Goyal D., Longo L.D., Clyman R.I. (2016). Microarray gene expression analysis in ovine ductus arteriosus during fetal development and birth transition. Pediatr. Res..

[14] Bokenkamp R., van Brempt R., van Munsteren J.C., van den Wijngaert I., de Hoogt R., Finos L., Goeman J., Groot A.C., Poelmann R.E., Blom N.A. (2014). Dlx1 and rgs5 in the ductus arteriosus: Vessel-specific genes identified by transcriptional profiling of laser-capture microdissected endothelial and smooth muscle cells. PLoS ONE.

[15] Shelton E.L., Ector G., Galindo C.L., Hooper C.W., Brown N., Wilkerson I., Pfaltzgraff E.R., Paria B.C., Cotton R.B., Stoller J.Z. (2014). Transcriptional profiling reveals ductus arteriosus-specific genes that regulate vascular tone. Physiol. Genom..

[16] Costa M., Barogi S., Socci N.D., Angeloni D., Maffei M., Baragatti B., Chiellini C., Grasso E., Coceani F. (2006). Gene expression in ductus arteriosus and aorta: Comparison of birth and oxygen effects. Physiol. Genom..

[17] Hsieh Y.T., Liu N.M., Ohmori E., Yokota T., Kajimura I., Akaike T., Ohshima T., Goda N., Minamisawa S. (2014). Transcription profiles of the ductus arteriosus in brown-norway rats with irregular elastic fiber formation. Circ. J..

[18] Yarboro M.T., Durbin M.D., Herington J.L., Shelton E.L., Zhang T., Ebby C.G., Stoller J.Z., Clyman R.I., Reese J. (2018). Transcriptional profiling of the ductus arteriosus: Comparison of rodent microarrays and human RNA sequencing. Semin. Perinatol..

[19] Mueller P.P., Drynda A., Goltz D., Hoehn R., Hauser H., Peuster M. (2009). Common signatures for gene expression in postnatal patients with patent arterial ducts and stented arteries. Cardiol. Young.

[20] Subramanian A., Tamayo P., Mootha V.K., Mukherjee S., Ebert B.L., Gillette M.A., Paulovich A., Pomeroy S.L., Golub T.R., Lander E.S. (2005). Gene set enrichment analysis: A knowledge-based approach for interpreting genome-wide expression profiles. Proc. Natl. Acad. Sci. USA.

[21] Saito J., Yokoyama U., Nicho N., Zheng Y.-W., Ichikawa Y., Ito S., Umemura M., Fujita T., Ito S., Taniguchi H. (2018). Tissue-type plasminogen activator contributes to remodeling of the rat ductus arteriosus. PLoS ONE.

[22] Ito S., Yokoyama U., Nakakoji T., Cooley M.A., Sasaki T., Hatano S., Kato Y., Saito J., Nicho N., Iwasaki S. (2020). Fibulin-1 Integrates Subendothelial Extracellular Matrices and Contributes to Anatomical Closure of the Ductus Arteriosus. Arterioscler. Thromb. Vasc. Biol..

[23] Blom M., Reis K., Heldin J., Kreuger J., Aspenstrom P. (2017). The atypical Rho GTPase RhoD is a regulator of actin cytoskeleton dynamics and directed cell migration. Exp. Cell Res..

[24] Kang Y., Kim J., Anderson J.P., Wu J., Gleim S.R., Kundu R.K., McLean D.L., Kim J.D., Park H., Jin S.W. (2013). Apelin-APJ signaling is a critical regulator of endothelial MEF2 activation in cardiovascular development. Circ. Res..

[25] Shimoda M., Yoshida H., Mizuno S., Hirozane T., Horiuchi K., Yoshino Y., Hara H., Kanai Y., Inoue S., Ishijima M. (2017). Hyaluronan-Binding Protein Involved in Hyaluronan Depolymerization Controls Endochondral Ossification through Hyaluronan Metabolism. Am. J. Pathol..

[26] Benso A., Broglio F., Marafetti L., Lucatello B., Seardo M.A., Granata R., Martina V., Papotti M., Muccioli G., Ghigo E. (2004). Ghrelin and Synthetic Growth Hormone Secretagogues are Cardioactive Molecules with Identities and Differences. Semin. Vasc. Med..

[27] Luo X., Liu J., Zhou H., Chen L. (2018). Apelin/APJ system: A critical regulator of vascular smooth muscle cell. J. Cell. Physiol..

[28] Scholler N., Hayden-Ledbetter M., Hellstrom K.E., Hellstrom I., Ledbetter J.A. (2001). Cd83 is an i-type lectin adhesion receptor that binds monocytes and a subset of activated cd8+ t cells [corrected]. J. Immunol..

[29] Khabibullin D., Medvetz D.A., Pinilla M., Hariharan V., Li C., Hergrueter A., Contreras M.L., Zhang E., Parkhitko A., Yu J.J. (2014). Folliculin regulates cell-cell adhesion, AMPK, and mTORC1 in a cell-type-specific manner in lung-derived cells. Physiol. Rep..

[30] Alba G.A., Samokhin A.O., Wang R.S., Zhang Y.Y., Wertheim B.M., Arons E., Greenfield E.A., Slingsby M.H.L., Ceglowski J.R., Haley K.J. (2021). NEDD9 is a Novel and Modifiable Mediator of Platelet-Endothelial Adhesion in the Pulmonary Circulation. Am. J. Respir. Crit. Care Med..

[31] Aquino J.B., Lallemend F., Marmigere F., Adameyko I., Golemis E.A., Ernfors P. (2009). The retinoic acid inducible Cas-family signaling protein Nedd9 regulates neural crest cell migration by modulating adhesion and actin dynamics. Neuroscience.

[32] Dieci E., Casati L., Pagani F., Celotti F., Sibilia V. (2014). Acylated and unacylated ghrelin protect MC3T3-E1 cells against tert-butyl hydroperoxide-induced oxidative injury: Pharmacological characterization of ghrelin receptor and possible epigenetic involvement. Amino Acids.

[33] Robertson I.B., Horiguchi M., Zilberberg L., Dabovic B., Hadjiolova K., Rifkin D.B. (2015). Latent tgf-beta-binding proteins. Matrix Biol..

[34] Zhou Y., Cashman T.J., Nevis K.R., Obregon P., Carney S.A., Liu Y., Gu A., Mosimann C., Sondalle S., Peterson R.E. (2011). Latent tgf-beta binding protein 3 identifies a second heart field in zebrafish. Nature.

[35] Huang J., Cheng L., Li J., Chen M., Zhou D., Lu M.M., Proweller A., Epstein J.A., Parmacek M.S. (2008). Myocardin regulates expression of contractile genes in smooth muscle cells and is required for closure of the ductus arteriosus in mice. J. Clin. Investig..

[36] Ivey K.N., Sutcliffe D., Richardson J., Clyman R.I., Garcia J.A., Srivastava D. (2008). Transcriptional regulation during development of the ductus arteriosus. Circ. Res..

[37] Feng X., Krebs L.T., Gridley T. (2010). Patent ductus arteriosus in mice with smooth muscle-specific Jag1 deletion. Development.

[38] Hohenauer T., Berking C., Schmidt A., Haferkamp S., Senft D., Kammerbauer C., Fraschka S., Graf S.A., Irmler M., Beckers J. (2013). The neural crest transcription factor brn3a is expressed in melanoma and required for cell cycle progression and survival. EMBO Mol. Med..

[39] Mi S., Wang P., Lin L. (2020). Mir-188-3p inhibits vascular smooth muscle cell proliferation and migration by targeting fibro-blast growth factor 1 (fgf1). Med. Sci. Monit..

[40] Heng W., Huang J.A., Wang Z.Y. (2012). Inhibition of Cellular Growth and Migration by Suppression of Endothelial Protein C Receptor (EPCR) in Lung Carcinoma Cells. Oncol. Res..

[41] Robb L., Hartley L., Wang C.C., Harvey R.P., Begley C.G. (1998). Musculin: A murine basic helix-loop-helix transcription factor gene expressed in embryonic skeletal muscle. Mech. Dev..

[42] Järveläinen H., Vernon R.B., Gooden M.D., Francki A., Lara S., Johnson P.Y., Kinsella M.G., Sage E.H., Wight T.N. (2004). Overexpression of Decorin by Rat Arterial Smooth Muscle Cells Enhances Contraction of Type I Collagen In Vitro. Arterioscler. Thromb. Vasc. Biol..

[43] Figueroa C.D., Marchant A., Novoa U., Forstermann U., Jarnagin K., Scholkens B., Muller-Esterl W. (2001). Differential distribution of bradykinin b(2) receptors in the rat and human cardiovascular system. Hypertension.

[44] Danielson K.G., Baribault H., Holmes D.F., Graham H., Kadler K.E., Iozzo R.V. (1997). Targeted Disruption of Decorin Leads to Abnormal Collagen Fibril Morphology and Skin Fragility. J. Cell Biol..

[45] Reinboth B., Hanssen E., Cleary E.G., Gibson M.A. (2002). Molecular interactions of biglycan and decorin with elastic fiber components: Biglycan forms a ternary complex with tropoelastin and microfibril-associated glycoprotein 1. J. Biol. Chem..

[46] Schussler O., Gharibeh L., Mootoosamy P., Murith N., Tien V., Rougemont A.L., Sologashvili T., Suuronen E., Lecarpentier Y., Ruel M. (2020). Cardiac Neural Crest Cells: Their Rhombomeric Specification, Migration, and Association with Heart and Great Vessel Anomalies. Cell. Mol. Neurobiol..

[47] Sawada H., Rateri D.L., Moorleghen J.J., Majesky M.W., Daugherty A. (2017). Smooth Muscle Cells Derived From Second Heart Field and Cardiac Neural Crest Reside in Spatially Distinct Domains in the Media of the Ascending Aorta—Brief Report. Arterioscler. Thromb. Vasc. Biol..

[48] Zhou Z., Wang J., Guo C., Chang W., Zhuang J., Zhu P., Li X. (2017). Temporally Distinct Six2 -Positive Second Heart Field Progenitors Regulate Mammalian Heart Development and Disease. Cell Rep..

[49] Wang X., Chen D., Chen K., Jubran A., Ramirez A., Astrof S. (2017). Endothelium in the pharyngeal arches 3, 4 and 6 is derived from the second heart field. Dev. Biol..

[50] Kim H.S., Aikawa M., Kimura K., Kuroo M., Nakahara K., Suzuki T., Katoh H., Okamoto E., Yazaki Y., Nagai R. (1993). Ductus arteriosus. Advanced differentiation of smooth muscle cells demonstrated by myosin heavy chain isoform expression in rabbits. Circulation.

[51] Schneider D.J., Moore J.W. (2006). Patent ductus arteriosus. Circulation.

[52] Bokenkamp R., DeRuiter M.C., van Munsteren C., de Groot A.C.G. (2010). Insights into the pathogenesis and genetic background of patency of the ductus arteriosus. Neonatology.

[53] Hajj H., Dagle J.M. (2012). Genetics of Patent Ductus Arteriosus Susceptibility and Treatment. Semin. Perinatol..

[54] Lewis T.R., Shelton E.L., Van Driest S.L., Kannankeril P.J., Reese J. (2018). Genetics of the patent ductus arteriosus (PDA) and pharmacogenetics of PDA treatment. Semin. Fetal Neonatal Med..

[55] Rose V., Izukawa T., Moes C.A. (1975). Syndromes of asplenia and polysplenia. A review of cardiac and non-cardiac malformations in 60 cases withspecial reference to diagnosis and prognosis. Br. Heart J..

[56] Raya A., Kawakami Y., Rodriguez-Esteban C., Buscher D., Koth C.M., Itoh T., Morita M., Raya R.M., Dubova I., Bessa J.G. (2003). Notch activity induces nodal expression and mediates the establishment of left-right asymmetry in vertebrate embryos. Genes. Dev..

[57] Krebs L.T., Iwai N., Nonaka S., Welsh I.C., Lan Y., Jiang R., Saijoh Y., O’Brien T.P., Hamada H., Gridley T. (2003). Notch signaling regulates left-right asymmetry determination by inducing nodal expression. Genes. Dev..

[58] De Angelis M.H., McIntyre J., Gossler A. (1997). Maintenance of somite borders in mice requires the delta homologue dii1. Nature.

[59] Baeten J.T., Jackson A.R., McHugh K.M., Lilly B. (2015). Loss of Notch2 and Notch3 in vascular smooth muscle causes patent ductus arteriosus. Genesis.

[60] Xue Y., Gao X., Lindsell C.E., Norton C.R., Chang B., Hicks C., Gendron-Maguire M., Rand E.B., Weinmaster G., Gridley T. (1999). Embryonic Lethality and Vascular Defects in Mice Lacking the Notch Ligand Jagged1. Hum. Mol. Genet..

[61] Kamath B.M., Spinner N.B., Emerick K.M., Chudley A.E., Booth C., Piccoli D.A., Krantz I.D. (2004). Vascular anomalies in alagille syndrome: A significant cause of morbidity and mortality. Circulation.

[62] Spinner N.B., Colliton R.P., Crosnier C., Krantz I.D., Hadchouel M., Meunier-Rotival M. (2001). Jagged1 mutations in alagille syndrome. Hum. Mutat..

[63] Clyman R.I., Mauray F., Roman C., Rudolph A.M., Heymann M.A., Maury F. (1980). Circulating prostaglandin E2 concentrations and patent ductus arteriosus in fetal and neonatal lambs. J. Pediatr..

[64] Smith G.C. (1998). The pharmacology of the ductus arteriosus. Pharmacol. Rev..

[65] Mitani Y., Takabayashi S., Sawada H., Ohashi H., Hayakawa H., Ikeyama Y., Imanaka-Yoshida K., Maruyama K., Shimpo H., Komada Y. (2007). Fate of the “opened” arterial duct: Lessons learned from bilateral pulmonary artery banding for hypoplastic left heart syndrome under the continuous infusion of prostaglandin E1. J. Thorac. Cardiovasc. Surg..

[66] De Groot A.C.G., Strengers J.L. (1988). Histopathology of the arterial duct (ductus arteriosus) with and without treatment with prostaglandin E1. Int. J. Cardiol..

