# Supplementary material for: Transcriptome Analysis Reveals Differential Gene Expression between the Closing Ductus Arteriosus and the Patent Ductus Arteriosus in Humans
Source: J Cardiovasc Dev Dis. 2021 Apr 16;8(4):45. doi: 10.3390/jcdd8040045 (PMC8073410; doi:10.3390/jcdd8040045)
Supplement: Supplementary file 1 [file jcdd-08-00045-s001.zip › jcdd-1155657-supplementary.pdf]

Table S1. Gene Ontology biological process terms (size > 300) that were significantly upregulated (FDR < 0.25) in the outer part of a longer-term PGE_1-treated human ductus arteriosus (DA) tissue (Case 4) compared to the tunica media of shorter-term PGE_1-treated DA tissues (Cases 2 and 3).

Gene Set Name	Size	NES	FDR *q*-Value	Rank at MAX
GO_ ORGANELLE_FISSION	431	1.46	0.071	7016
GO_NEGATIVE_REGULATION_OF_CELL_CYCLE_PROCESS	315	1.38	0.181	7124
GO_MICROTUBULE_BASED_MOVEMENT	314	1.38	0.132	7727
GO_MICROTUBULE_CYTOSKELETON_ORGANIZATION	516	1.37	0.115	5962
GO_MICROTUBULE_BASED_PROCESS	740	1.34	0.149	5962
GO_REGULATION_OF_CELL_CYCLE_PHASE_TRANSITION	421	1.30	0.249	6256
GO_REGULATION_OF_CELL_CYCLE_PROCESS	705	1.29	0.234	6958
GO_CELL_CYCLE_PHASE_TRANSITION	578	1.29	0.212	5961
GO_CELL_DIVISION	546	1.27	0.239	5612

Abbreviations: NES, normalized enrichment score; FDR, false discovery rate

Table S2. Twenty genes that overlapped and were enriched (> 8-fold) in the outer part of a longer-term PGE_1-treated DA tissue (Case 4) compared to the IT of shorter-term PGE_1-treated DA tissues (Cases 2 and 3).

Gene Name	Description	Fold Change	
		Case 1 vs. Case 2	Case 1 vs. Case 3
CLEC3A	C-type lectin domain family 3 member A	19.5	187.6
ATP6V1G3	ATPase H+ transporting V1 subunit G3	67.6	62.6
SAA4	serum amyloid A4, constitutive	56.5	59.0
BEX1	brain expressed X-linked 1	74.4	10.3
PCP4	Purkinje cell protein 4	71.7	10.2
SPATA17	spermatogenesis associated 17	35.4	32.2
RGS13	regulator of G-protein signaling 13	32.7	30.1
ZNF837	zinc finger protein 837	10.4	29.2
RNF17	ring finger protein 17	26.9	8.4
KIAA1024L	PREDICTED: *KIAA1024*-like	17.5	17.3
LCMT2	leucine carboxyl methyltransferase 2	15.8	17.7
ATP8A2	ATPase phospholipid transporting 8A2	14.9	13.7
EPPIN	epididymal peptidase inhibitor	14.1	13.1
ARRB1	arrestin beta 1	18.0	8.9
CHRDL2	chordin-like 2	17.7	8.8
CIAPIN1	cytokine induced apoptosis inhibitor 1	10.4	14.0
VTCN1	V-set domain containing T cell activation inhibitor 1	12.6	8.6
FEM1A	fem-1 homolog A	10.3	10.8
DSCC1	DNA replication and sister chromatid cohesion 1	8.8	9.7
VIT	vitrin	9.5	8.4

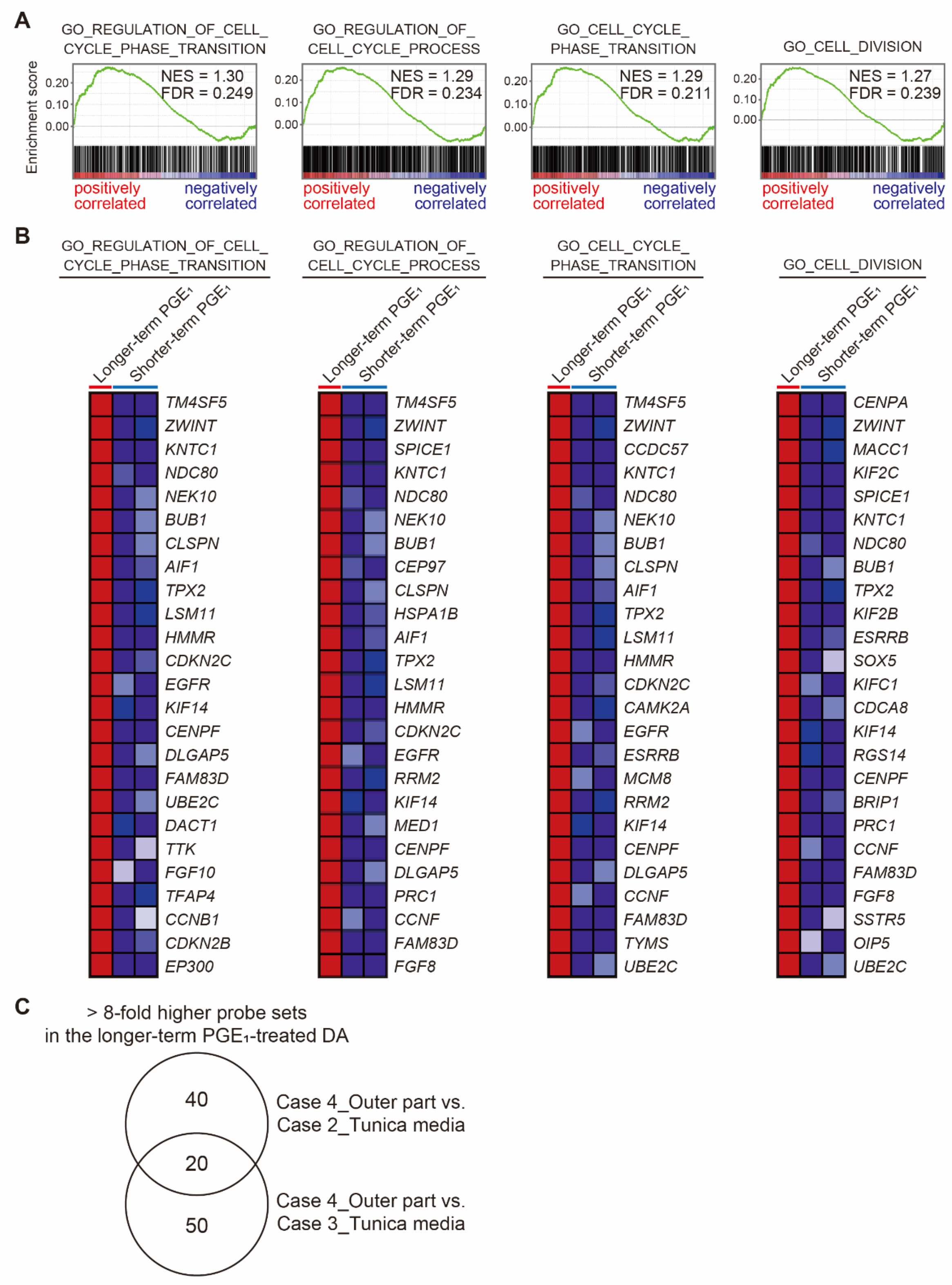

Figure S1. Differential gene expression between the longer-term PGE1-treated human ductus arteriosus (DA)

tissue (Case 4) and the shorter-term PGE_1-treated DA tissues (Cases 2 and 3). (**A**) Gene-set enrichment analyses (GSEAs) revealed positive correlations between the outer part of the long-term PGE_1-treated DA tissue and cell-cycle related genes. (**B**) The top 25 genes that comprise the leading edge of the enrichment score in (**A**) are shown as a heatmap. (**C**) A Venn diagram shows the numbers of probe sets that were highly expressed (> 8-fold) in the outer part of longer-term PGE_1-treated DA tissue (Case 4) compared to the tunica media of shorter-term PGE_1-treated DA tissues (Cases 2 and 3). NES, normalized enrichment score; FDR, false discovery rate. .
